# Role of the splicing factor SRSF4 in cisplatin-induced modifications of pre-mRNA splicing and apoptosis

**DOI:** 10.1186/s12885-015-1259-0

**Published:** 2015-04-07

**Authors:** Maude Gabriel, Yves Delforge, Adeline Deward, Yvette Habraken, Benoit Hennuy, Jacques Piette, Roscoe Klinck, Benoit Chabot, Alain Colige, Charles Lambert

**Affiliations:** 1 Laboratory of Connective Tissues Biology, GIGA-Cancer, University of Liège, avenue de l’Hôpital 1, 4000 Liège, Belgium; 2 Laboratory of Virology and Immunology, GIGA-Signal Transduction, GIGA B34, University of Liège, avenue de l’Hôpital 1, 4000 Liège, Belgium; 3 GIGA Genomics Platform, University of Liège, avenue de l’Hôpital 1, 4000 Liège, Belgium; 4 Laboratory of Functional Genomics and Department of Microbiology and Infectiology, Faculty of Medicine and Health Sciences, Université de Sherbrooke, Sherbrooke, Québec Canada

**Keywords:** Cancer therapy, Alternative splicing, PI3K, Apoptosis, Drug efficiency, Cisplatin, SRSF4

## Abstract

**Background:**

Modification of splicing by chemotherapeutic drugs has usually been evaluated on a limited number of pre-mRNAs selected for their recognized or potential importance in cell proliferation or apoptosis. However, the pathways linking splicing alterations to the efficiency of cancer therapy remain unclear.

**Methods:**

Next-generation sequencing was used to analyse the transcriptome of breast carcinoma cells treated by cisplatin. Pharmacological inhibitors, RNA interference, cells deficient in specific signalling pathways, RT-PCR and FACS analysis were used to investigate how the anti-cancer drug cisplatin affected alternative splicing and the cell death pathway.

**Results:**

We identified 717 splicing events affected by cisplatin, including 245 events involving cassette exons. Gene ontology analysis indicates that cell cycle, mRNA processing and pre-mRNA splicing were the main pathways affected. Importantly, the cisplatin–induced splicing alterations required class I PI3Ks P110β but not components such as ATM, ATR and p53 that are involved in the DNA damage response. The siRNA-mediated depletion of the splicing regulator SRSF4, but not SRSF6, expression abrogated many of the splicing alterations as well as cell death induced by cisplatin.

**Conclusion:**

Many of the splicing alterations induced by cisplatin are caused by SRSF4 and they contribute to apoptosis in a process requires class I PI3K.

**Electronic supplementary material:**

The online version of this article (doi:10.1186/s12885-015-1259-0) contains supplementary material, which is available to authorized users.

## Background

Chemotherapy with platinum-based compounds is used extensively for the treatment of a wide range of solid tumours, including breast cancers resistant to first line therapy, ovarian, non-small cell lung, testis, endometrial, head and neck and colorectal cancers. Cisplatin (cis-diamine platinum (II) dichloride), the founding member of this class of agents, covalently binds to DNA and induces the formation of bulky DNA adducts consisting of intra-strand cross-links preferentially formed between adjacent guanine residues and, to a lower extent, inter-strand DNA lesions [1,2]. Cell toxicity is linked to these adducts that interfere with DNA replication and transcription. Intra-strand cross-links are mainly processed by removal of platinum adducts via the nucleotide excision repair, and inter-strand cross-links are removed via nucleotide excision repair, translesion polymerase and homologous recombination. Cisplatin activates various signalling pathways that include the DNA damage response (DDR) and the PI3K-Akt pathways [1]. The DDR pathway detects and corrects DNA defects. However, when alterations are too numerous or too severe, cells are committed to death and eliminated. The DDR machinery relies on the activity of three enzymes that belong to the phosphatidyl inositol-3 kinases (PI3K) family: DNA-protein kinase (DNA-PK), Ataxia and Telangectasia Mutated (ATM) and Ataxia Telangiectasia and Rad 3-Related (ATR) [3]. These kinases trigger specific and overlapping cascades of signalling events that result in cell cycle arrest, DNA repair or cell death [4].

Alternative splicing (AS) occurs in more than 90% of multi-exons primary transcripts [4,5]. Proteins produced through AS can have markedly different and sometimes opposite functions, as exemplified by a number of factors involved in apoptosis or cell survival [6]. In other instances, AS controls the level of proteins by producing transcripts carrying premature termination codons that are degraded by non-sense mediated RNA decay (NMD) [7]. Splicing decisions result from an interplay between highly degenerated cis-acting sequences and a large number of trans-acting factors that include the arginine- and serine-rich proteins (SR-proteins) and the heterogenous nuclear ribonucleoproteins (hnRNPs) families [8]. The participation of these factors in splicing control is often regulated by post-translational modifications such as phosphorylation and acetylation which affect their localisation and their interaction with other proteins [8].

Aberrant AS occurs in cancer and a growing number of studies have reported a functional link between splicing anomalies and the evolution of the disease [9-12]. Several groups, including ours, have shown that chemotherapeutic drugs can affect the AS of a large number of transcripts [13-16]. However, the impact of these changes on the cancer cell is still poorly understood. Here, we analyse the transcriptome of cisplatin-treated cancer cells, and use AS changes to identify pathways that link cisplatin with the cellular response.

## Methods

### Cell culture, authentication, reagent and survival assay

MCF7, MDA-MB-231, HT1080, BT549, RD, HDF1 and HDF2, MG-63, MSU and AT5BIVA (deficient in ATM, Coriell Cell Repository, Camden, NJ, USA) cells were cultured in Dulbecco’s Modified Eagle’s Medium (DMEM, Lonza, Verviers, Belgium) supplemented with non-essential amino-acids (NEAA) (1%), penicillin and streptomycine (1%), gentamycin (0.1%), fungizone (0.1%) and 10% FCS (Lonza). Ishikawa cells (human endometrial adenocarcinoma cell line) were cultured in RPMI 1640-glutamax (Lonza) supplemented with NEAA (1%), sodium pyruvate (1%), penicillin and streptomycine (1%), fungizone (0.1%) and 10% FCS, GM09607 cells (deficient in ATM, Coriell Cell Repository) in EMEM (Lonza) supplemented with 10% FCS and 1% NEAA, and MO59J cells (glioblastoma cell line, deficient in the catalytic subunit of DNA-PK) in DMEM/F12 supplemented as DMEM.

The study conforms to the principles outlined in the Declaration of Helsinki and was approved by the ethic committee of Liège University Hospital (B707201110973).

MCF7 and Ishikawa cells were authenticated by DSMZ (Braunschweig, Germany). Although no authentication of the other cell lines was made, the deficiency in ATM of GM09607 and AT5BIVA was ascertained by western blotting, and that of p53 in MG-63 was confirmed by RT-PCR.

Cisplatin (cis-diamine platinum (II) dichloride), wortmannin, caffein, and triciribine were from Sigma-Aldrich (St-Louis, MO, USA), oxaliplatin from Santa Cruz Biotechnology (Santa Cruz, CA, USA), ATM kinase inhibitor from Calbiochem EMD biosciences (La Jolla, CA, USA), NU7026 from Merck Millipore (Darmstadt, Germany), TGX221, IC87114 and MK2206 from Selleckchem (Munich, Germany) and PX866 from LC Laboratories (Woburn, MA, USA).

Cell survival and apoptosis/necrosis were measured, respectively, by trypan blue exclusion in blind tests and by FACS analysis as described in [17].

### RNA isolation, RT-PCR and RT-qPCR

RNAs were purified from cultured cells using the High Pure RNA isolation kit (Roche, Mannheim, Germany) and quantified by spectrometry. Gene expression was measured by RT-qPCR. Details according to the Minimum Information for Quantitative RT-PCR Experiment (MIQE) guidelines [18], are given in Additional files 1 and 2. For analysis of exon inclusion/exclusion, primers were chosen on exons surrounding the sequences potentially alternatively spliced. Primers, protocols and amplification products sizes are detailed in Additional file 2. Splice variants were discriminated by electrophoresis as described [17].

### RNA sequencing analysis

RNA libraries and sequencing were performed on total RNA samples at the GIGA Genomics facility, University of Liège, Belgium. The quality of RNA was checked with BioAnalyser 2100 (Agilent technologies, CA, USA) that indicated a RQI score >8. The libraries were prepared with Truseq® mRNA Sample Prep kit (Illumina, CA, USA) from 1 microgram of total RNA following manufacturer’s instructions. mRNAs were isolated by poly-A selection and fragmented (8 minutes at 94°C). Fragmented mRNAs (around 170 nucleotide-long in average) were used for reverse-transcription in the presence of Superscript II (Invitrogen, Oregon, USA) and random primers. After second strand synthesis, end-repair, A-tailing and purification, the double strand cDNA fragments were ligated to Truseq® adapters containing the index sequences. Fifteen cycles of PCR in the presence of dedicated PCR primers and PCR master mix were applied to generate the final libraries. Libraries were sequenced in pair-end sequencing runs on the Illumina GAIIx in multiplexed 2 × 76 base protocols. The raw data was generated through CASAVA 1.6 suite (Illumina, CA, USA). TopHat (http://ccb.jhu.edu/software/tophat/index.shtml) software was used to align RNA-Seq reads to the reference genome (hg19, UCSA) and discover transcript splice sites. Cufflinks (http://cole-trapnelllab.github.io/cufflinks/) used the resulting alignment files to quantify the gene expression levels, identify up- and down-regulated transcripts and find the alternative splice junctions.

SpliceSeq(1.2) (http://bioinformatics.mdanderson.org/main/SpliceSeq:Overview) was used for a focused AS analysis. Using alignment database and Bowtie, SpliceSeq aligns reads from RNA-Seq data to a reference collection of splice variants [19,20].

### Gene prioritarization

Lists of genes modulated in term of expression and splicing were imported in the ToppGene Suite for analysis [21].

### Antibodies and Western blotting

Antibodies directed against Akt, phospho-Akt (ser473) and β-actin were purchased from Cell Signalling (Beverly, MA, USA). Cells were lysed in Laemmli buffer containing 50 mM DTT. Lysates were briefly sonicated, incubated at 65°C for 15 min and analyzed by SDS-PAGE. Proteins were electroblotted and detected as described in [17]. Probing of β-actin was performed as a control of protein loading.

### siRNA transfection

SMARTpool siGENOME (Dharmacon by Thermo Fisher Scientific, Lafayette, CO, USA), consisting of four siRNA duplexes, were used to target SRSF4 and SRSF6 mRNA. siRNA targeting ATR were from Ambion (Life technologies). The 5′-UUGCAUACAGGACUCGUUATT-3′ and 5′-UAACGAGUCCUGUAUGCAATT-3′ oligoribonucleotides were used as control siRNA (siSCR) that does not target any known human transcript [22]. Cells were transfected by siRNAs as previously described [23].

### Statistics

The means and standard deviation were calculated from three or four independent experiments. The significance of differences was determined using *t*-test or ratio paired *t*-test of Student.

## Results

### Cisplatin alters alternative splicing

**Figure 1:**
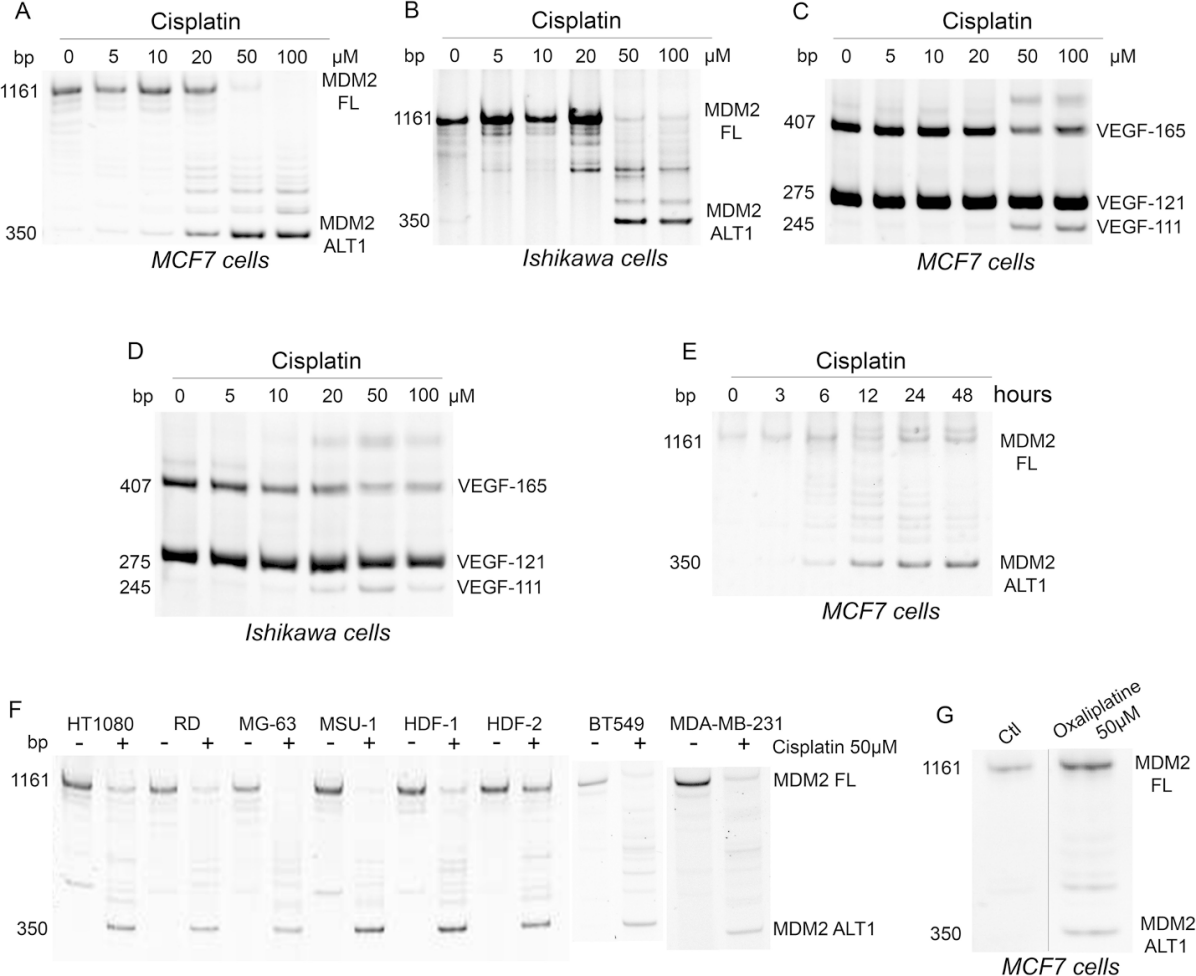
**Platinum-based chemotherapeutic agents affect MDM2 and VEGF pre-mRNA splicing. A**-**D**: MCF7 and Ishikawa cells were treated with the indicated concentrations of cisplatin and harvested after 24 hours. E: MCF7 cells treated with cisplatin (50 μM) were harvested at the indicated times. **F**: The indicated cells were treated with cisplatin (50 μM) for 24 hours. **G**: MCF7 cells were treated with oxaliplatin at 50 μM and harvested after 24 hours. Analysis of the splicing of MDM2 **(A,B,E-G)** or of VEGF-A **(C,D)** transcripts was performed by end-point RT-PCR and acrylamide gel electrophoresis as detailed in Methods. Illustrated gels are representative of three independent experiments. FL: Full Length; ALT1: Splice variant of MDM2.

In vitro treatment of cells by cisplatin induces alterations of splicing in various transcripts [15,24,25]. Following treatment of MCF7 and Ishikawa cell lines with cisplatin (Figure 1A-B), the RT-PCR analysis of MDM2, a negative regulator of p53, showed a reduction in the full length product and the appearance of smaller splicing variants. The smallest variant had the expected size of MDM2-ALT1 splice variant. This splicing shift was maximal at 50 – 100 μM of cisplatin (Figure 1A-B) and after 24 – 48 hours (Figure 1E). A similar dose-dependent shift was obtained with VEGF where cisplatin decreased the expression of VEGF-165 and concomitantly increased the production of the VEGF-111 splice variant (Figure 1C-D). Similar alterations of splicing of MDM2 were also observed in MDA-MB-231 (breast adenosarcoma), BT549 (breast carcinoma), HT1080 (fibrosarcoma), RD (rhabdomyosarcoma), MG-63 (osteosarcoma), MSU (fibrobastic cell line) and HDF1 and 2 (primary dermal fibroblasts) cells treated with 50 μM cisplatin for 24 hours (Figure 1F). The cisplatin analog oxaliplatin induced similar effects on MDM2 splicing, suggesting that this splicing alteration is generalized to platinum-based agents (Figure 1G).

### Deep sequencing

Poly A+ RNA from MCF7 cells untreated or treated with 50 μM cisplatin for 24 hours was isolated and prepared for next-generation sequencing analysis. No significant cell death over untreated samples was noted in these conditions (as measured by trypan blue exclusion). The average number of reads approached or exceeded 20 millions in both samples. Alignment of transcripts to the genome indicated that 16733 and 16969 genes were expressed in the control and cisplatin-treated samples, respectively. Sequencing data are available in the ArrayExpress database (www.ebi.ac.uk/arrayexpress) under accession number E-MTAB-2663. The global gene expression in the two conditions was highly correlated, with a Pearson correlation coefficient = 0.835 (p = 0.000000).

### Effect of cisplatin on gene expression

For differential gene expression, the following filters were applied: absolute fold change >2 and q-value < 0.05. Five hundred fifty-three genes were regulated (111 up and 442 down, Additional file 3). The top 20 up-regulated and down-regulated genes are listed in Table 1 with SERPINB5 (126×) and GPHN (159×) being first in each category, respectively. The expression of a panel of genes commonly used as calibrators was not significantly affected (GAPDH: 1.20; ACTB: 0.79; ACTG: 0.93; PPIA (cyclophylin A): 0.94; PPIB (cyclophylin B): 1.07). RT-qPCR was performed to confirm the expression level of 9 up-, down- or non-regulated genes on the samples used for RNA-Seq. Selected genes were either conventional calibrators (GAPDH, ACTB, β2M) or encoded proto-oncogenes (MYB, SERPINB5, JAK2), anti-oncogenes (BRCA1, RB1) and a factor regulating apoptosis (FAS). RT-qPCR analysis correlated with RNA-seq data with a Pearson coefficient of 0.98 (p = 0.000004), validating the RNA-seq data. Moreover, these changes in gene expression noted by RT-qPCR were confirmed in three independent experiments using MCF7 cells, and in two independent experiments in Ishikawa cells, indicating that the changes are reproducible and not restricted to MCF7 cells.

**Table 1:** **Top twenty up- and down-regulated genes by cisplatin in MCF7 cells**

Gene	Name	Fold_change	q_value
SERPINB5	serpin peptidase inhibitor, Clade B (Ovalbumin), member 5	126	1.54E-08
POU3F1	POU class 3 homeobox 1	105	6.15E-03
NKX1-2	NK1 homeobox 2	75	3.63E-02
LAMP3	lysosomal-associated membrane protein 3	63	2.28E-02
ATF3	activating transcription factor 3	51	3.58E-07
GADD45A	growth arrest and DNA-damage-inducible, alpha	51	1.51E-07
HBEGF	heparin-binding EGF-like growth factor	44	8.31E-06
HES2	hairy and enhancer of split 2 (Drosophila)	41	3.59E-05
NGFR	nerve growth factor receptor	41	4.80E-04
SNAI1	snail family zinc finger 1	32	1.82E-03
GPR3	G protein-coupled receptor 3	32	4.17E-03
GPR172B	solute carrier family 52, riboflavin transporter, member 1	29	2.38E-03
PTAFR	platelet-activating factor receptor	28	2.45E-04
PRODH	proline dehydrogenase (oxidase) 1	27	2.92E-02
C5orf4	chromosome 13 open reading frame, human	27	2.07E-03
PMAIP1	phorbol-12-myristate-13-acetate-induced protein 1	22	1.26E-06
HAP1	huntingtin-associated protein 1	21	3.08E-02
FAS	TNF receptor superfamily member 6	21	4.92E-05
GUCA1B	guanylate cyclase activator 1B (retina)	20	4.71E-03
LIF	leukemia inhibitory factor	19	5.31E-05
ROBO1	roundabout, axon guidance receptor, homolog 1	−46	2.45E-06
NEGR1	neuronal growth regulator 1	−48	7.78E-04
EYA4	eyes absent homolog 4	−49	3.90E-03
CADPS2	Ca++ − dependent secretion activator 2	−49	7.97E-03
SLCO3A1	solute carrier organic anion transporter family, member 3A1	−49	3.07E-02
SAMD12	sterile alpha motif domain containing 12	−50	4.74E-04
NFIA	nuclear factor I/A	−53	2.38E-04
SULF1	sulfatase 1	−55	6.43E-04
MAGI1	membrane associated guanylate kinase, WW and PDZ domain containing 1	−56	5.79E-05
HS6ST3	heparan sulfate 6-O-sulfotransferase 3	−62	1.80E-04
PLCH1	phospholipase C, eta 1	−62	2.47E-03
PPP1R9A	protein phosphatase 1, regulatory subunit 9A	−67	1.24E-03
KCNJ8	potassium inwardly-rectifying channel, subfamily J, member 8	−67	3.33E-04
MLLT3	myeloid/lymphoid or mixed-lineage leukemia translocated to, 3	−67	1.50E-02
PLXDC2	plexin domain containing 2	−70	2.52E-04
SEMA5A	semaphorin 5A	−75	1.50E-04
ERBB4	v-erb-a erythroblastic leukemia viral oncogene homolog 4 (avian)	−81	7.36E-04
LTBP1	latent transforming growth factor beta binding protein 1	−94	3.39E-05
TIAM1	T-cell lymphoma invasion and metastasis 1	−146	8.42E-03
GPHN	Gephyrin	−159	3.85E-05

Fold change expression in cisplatin-treated (50 μM, 24 hours) samples relative to control and q-values as measured by RNA-seq are indicated.

Gene ontology analysis was performed using the ToppFun Suite software. Significantly (p < 0.05) affected biological processes were identified (Table 2). Many genes regulated by cisplatin belong to two main groups: cell cycle and proliferation. Surprisingly, neurogenesis also appeared as a regulated category.

**Table 2:** **Significantly enriched biological processes affected by cisplatin**

Pathway	P-value	Modulated genes in the treated cells	Total genes in the pathway
*Expression*			
Cell cycle	0.001083	85	1399
Enzyme linked receptor protein signaling pathway	0.002002	62	886
Regulation of cell proliferation	0.002877	75	1189
Regulation of cell cycle	0.007228	54	741
Negative regulation of cell cycle	0.02095	39	456
Cell cycle process	0.03652	67	1070
Neurogenesis	0.03869	70	1142
*Splicing*			
mRNA metabolic process	3.89E-12	69	635
RNA splicing	2.102E-10	46	328
mRNA processing	5.512E-10	51	408
RNA processing	1.002E-09	67	671
RNA splicing, via transesterification reactions	7.631E-07	33	219
Nuclear mRNA splicing, via spliceosome	1.935E-05	32	213
RNA splicing, via transesterification reactions with bulged adenosine as nucleophile	1.935E-05	32	213
Cell cycle	0.01964	89	1455

Genes that were regulated by more than 2-fold (Expression) or transcripts alternatively spliced (Splicing) by cisplatin were analysed by the ToppFun Suite software. The identified biological processes are indicated.

ToppFun identified genes matching annotations for transcription factors PITX2 (38 genes), E2F (18 genes, Table 3) and FOXF2 (19 genes). For genes matching with PITX2 and FOX2, no significant difference in the proportion of up- and down-regulated genes (versus total numbers of up- and down-regulated genes, respectively) was observed. In contrast, the 18 genes matching with E2F were all down-regulated. As the expression of E2F transcription factors themselves was not significantly changed, this suggests that cisplatin may affect their activity.

**Table 3:** **Genes regulated by cisplatin and matching annotations for transcription factors E2F**

Name	Fold change	q-value
HS6ST3	−59.7	0.000
SEMA5A	−73.5	0.000
STAG1	−18.4	0.000
JPH1	−17.1	0.001
EFNA5	−39.4	0.001
MSH2	−6.5	0.002
SLC38A1	−5.7	0.002
CBX5	−3.7	0.017
NASP	−6.1	0.020
DNMT1	−4.6	0.030
SLCO3A1	−48.5	0.031
MCM6	−4.3	0.035
MCM3	−5.7	0.036
RPS6KA5	−8.6	0.038
FANCD2	−4.6	0.047
USP37	−5.7	0.047
CLSPN	−4.6	0.050
CDC6	−4.3	0.412

The fold change and q-value are indicated.

The list of genes regulated by cisplatin was compared to lists of oncogenes (http://www.uniprot.org/uniprot/?query=keyword:KW-0656) and tumor suppressors (http://www.uniprot.org/uniprot/?query=keyword:KW-0043). Data showed that cisplatin reduced the level of some tumor suppressor genes and of oncogenes while inducing others (Table 4). Strikingly, the expression of the two AP-1 members FOS and JUN was found to be increased.

**Table 4:** **Cisplatin regulates the expression of tumor suppressors and oncogenes**

Name	Fold change	q-value
*Tumor suppressors*		
SERPINB5	128.0	0.000
TP53INP1	9.2	0.000
SULF1	−55.7	0.001
ERBB4	−78.8	0.001
BUB1B	−21.1	0.002
MAFB	10.6	0.004
STARD13	−22.6	0.004
HIPK2	−13.0	0.004
SASH1	−11.3	0.009
MTUS1	−6.1	0.027
BRCA1	−7.0	0.031
ST7	−27.9	0.033
RB1	−4.3	0.035
IRF1	4.9	0.040
TP63	−34.3	0.042
FANCD2	−4.6	0.047
*Oncogenes*		
NCOA1	−13.0	0.001
MAFB	10.6	0.004
FOS	8.6	0.004
JAK2	−11.3	0.005
PRKCA	−12.1	0.006
GMPS	−5.7	0.011
MYB	−21.1	0.013
MLLT3	−68.6	0.015
AKAP13	−6.1	0.019
JUN	4.6	0.028
MCF2L	−6.5	0.035

The fold change and p-value are indicated. The list of the genes regulated by cisplatin was compared to lists of oncogenes (http://www.uniprot.org/uniprot/?query=keyword:KW-0656) and tumours suppressors (http://www.uniprot.org/uniprot/?query=keyword:KW-0043).

### Effect of cisplatin on post-transcriptional events

Potential modifications of splicing by cisplatin were investigated from the RNA-seq data. The SpliceSeq software identified 717 AS events occurring in 619 primary transcripts (Additional file 4). Only 5 genes (UGDH, SLC38A1, RETSAT, PDE8A, NASP) (0.44%) were affected simultaneously at transcriptional and post-transcriptional levels. Changes in splicing were grouped based on the type of events being affected: 79 changes involved cassette exon inclusion events, 166 were cassette exon exclusion events (of which 49% were not annotated as alternative exons in NCBI), 243 changes affected alternative 5′ or 3′ splice site selection events, 144 involved alternative promoters, 83 indicated alternative terminations and 2 were splicing changes attributed to mutually exclusive exon. Significantly affected biological processes identified by ToppFun Suite software on affected genes were “RNA splicing and processing” and “cell cycle” (Table 2).

**Figure 2:**
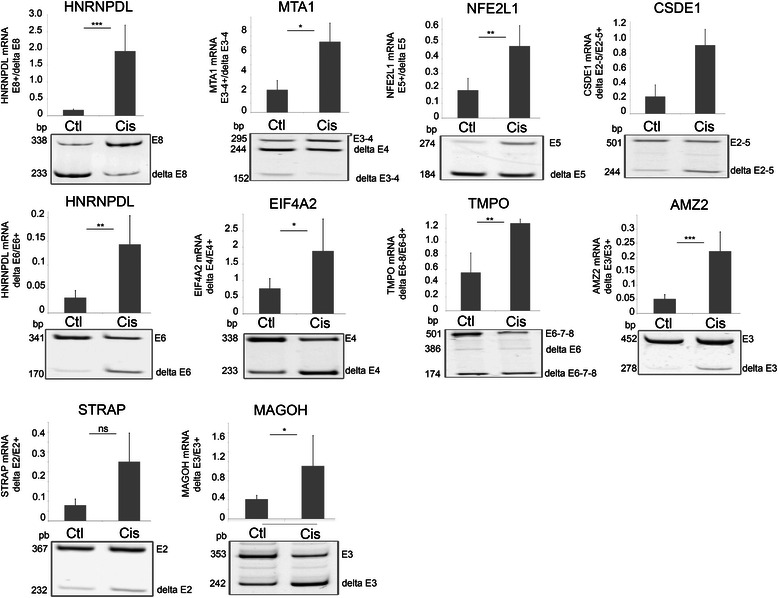
**Analysis of selected splicing events modified by cisplatin in MCF7 cells.** End-point RT-PCR and analysis of amplification products by acrylamide gel electrophoresis were performed on control and cisplatin (50 μM, 24 hours)-treated MCF7 cells to validate inclusion/exclusion events detected by RNA-Seq and SpliceSeq analysis. Cisplatin-treatment induced the inclusion of HNRNPDL exon 8 (E8; ***p = 0.0008), MTA1 exons 3–4 (E3-4; *p = 0.047) and NFE2L1 exon 5 (E5; **p = 0.008), and the exclusions of CSDE1 exons 2–4 (delta E2-4; ***p = 0.00003), HNRNPDL exon 6 (delta E6; **p = 0.0045), EIF4A2 exon 4 (delta E4; * = 0.02), TMPO exons 6–8 (delta E6-8; **p = 0.009), AMZ2 exon 3 (delta E3; ***p = 0.001), STRAP exon 2 (delta E2; p = 0.059) and MAGOH exon 3 (delta E3; * = 0.04). Graphs show the mean and SD and are representative of at least 3 independent experiments.

For validation purpose, 16 splicing events identified by SpliceSeq as affected by cisplatin were evaluated by RT-PCR. These events were chosen such as to cover the range of AltSplice RPKM (reads per kilobase per million reads) values from 0 to 40. RT-PCR confirmed the alternative splicing of the ten exon skipping events and four of the six exon inclusion events in MCF7 and Ishikawa cells, indicating a good concordance with RefSeq data (drawings of nine splicing events in MCF-7 cells are shown in Figure 2).

### PI3K pathway, but not DNA damage response and p53, is involved in the alteration of splicing by cisplatin

**Figure 3:**
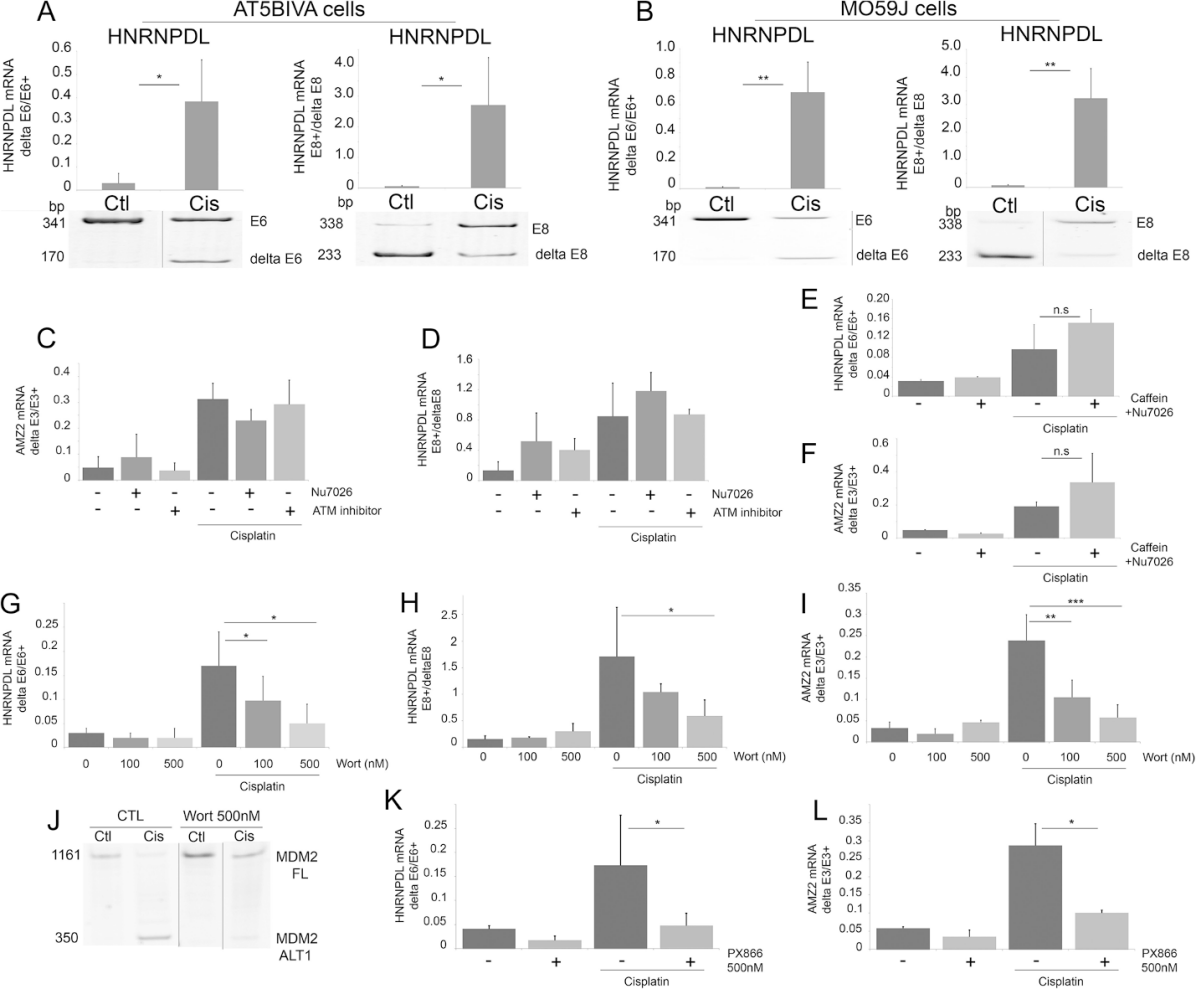
**Lack of contribution of ATM, ATR and DNA-PK pathways in cisplatin-induced splicing.** AT5BIVA (ATM deficient, **A)** and MO59J (DNA-PK deficient, **B)** cells were treated with cisplatin (50 μM, 24 hours) and analysed for alternative splicing events in HRNPDL pre-mRNA. (*p ≤ 0.05; **p ≤ 0.01); **C**-**D**: MCF7 cells were treated with ATM inhibitor (50 μM) or DNA-PK inhibitor (NU7026; 25 μM) three hours prior to treatment with cisplatin (50 μM for 24 hours); **E**-**F**: MCF7 cells were pre-treated with caffeine (5 mM) and with DNA-PK inhibitor (NU7026; 25 μM) for three hours prior to treatment with cisplatin (50 μM) (**E**: HNRNPDL-E6 p = 0.13; **F**: AMZ2: p = 0.49). **G**-**L**: MCF7 cells were treated with wortmannin (100 nM and 500 nM) or PX866 (500 nM), three hours prior to treatment with cisplatin (50 μM, 24 hours). Modifications of alternative splicing were evaluated for **G**: HNRNPDL-E6 *p = 0.02; **H**: HNRNPDL-E8 *p = 0.02; **I**: AMZ2 **p = 0.004, ***p = 0.0005; **J**: MDM2. Similar modification was observed with PX866 and illustrated for K: HNRNPDL-E6 *p = 0.05; **L**: AMZ2 *p = 0.02. Alternative splicing was evaluated by end-point RT-PCR and acrylamide gel electrophoresis. Each bar shows the mean with SD of at least three independent experiments.

As cisplatin induces DNA damage, the contribution of three main actors of the DDR pathway (ATM, ATR and DNA-PK) in cisplatin-induced exon inclusion/exclusion was investigated. Cisplatin affected the splicing in ATM-deficient AT5BIVA cells (illustrated for HNRNPDL exon 6 exclusion and exon 8 inclusion in Figure 3A) and GM9607 cells (not illustrated). Similarly to its effect in MCF7 cells (Figure 2), identical data were also found in MO59J cells that lack the catalytic subunit of DNA-PK (Figure 3B), and in MCF7 cells transfected with a siRNA targeting ATR (not shown). Moreover, specific inhibition of ATM (using ATM kinase inhibitor) or of DNA-PK (using NU7026) did not reverse the splicing induced by cisplatin in MCF7 cells (Figure 3C-D). Combined inhibition experiments were performed to evaluate whether the three DDR members might functionally compensate each other. Inhibition of ATM and ATR (using caffeine) and DNA-PK (using NU7026) failed to reverse the splicing induced by cisplatin (Figure 3E-F). The same results were obtained in MO59J cells (deficient in DNA-PK activity) treated with caffeine (not shown). Together, these data strongly suggest that the DDR does not participate in the splicing change of HNRNPDLe6 and AMZ2 induced by cisplatin. The DNA damage-activated protein p53 is similarly not involved since cisplatin induces an alteration of MDM2 splicing in the p53-deficient cell line MG-63 (Figure 1F).

**Figure 4:**
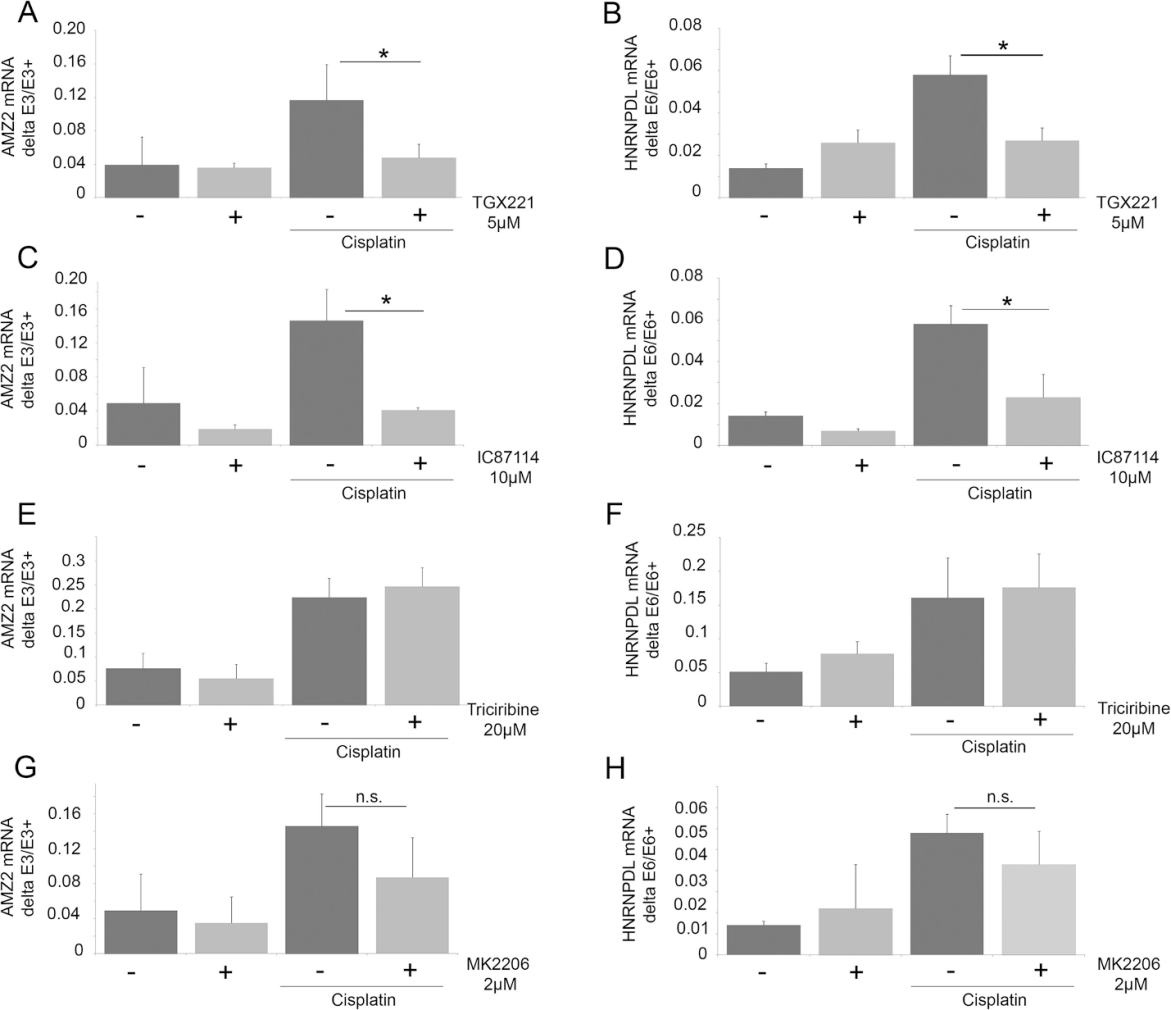
**Involvement of PI3K pathway, but not Akt, in cisplatin-induced splicing. A**-**F**: MCF7 cells were treated with TGX221 (5 μM; **A**-**B**) or IC87114 (10 μM; **C**-**D**) three hours prior to treatment with cisplatin (50 μM, 24 hours). **E**-**H**: MCF7 cells were treated with triciribine (20 μM) or MK2206 (2 μM) three hours before cisplatin treatment (50 μM; 24 hours). Alternative splicing of exon 6 of HNRNPDL **(B,D,F,H)** and exon 3 of AMZ2 **(A,C,E,G)** was evaluated by RT-PCR. RT-PCR products were fractionated by gel electrophoresis.

As cisplatin is also known to activate PI3K in several cell types [26], the implication of the PI3Ks in the AS changes induced by cisplatin was evaluated. MCF7 cells were pre-treated with wortmannin three hours prior to adding cisplatin. At the concentrations used, wortmannin inhibits class I and III PI3Ks as well as PI3KC2b, but not ATM, ATR, DNA-PK. Dose-dependent inhibition of the effect of cisplatin on the splicing events was observed (Figure 3G-J). A similar impact was observed with the wortmannin derivative PX866 (Figure 3K-L). To gain further insights into the identity of PI3Ks involved, inhibitors specifically targeting the class I PI3Ks (TGX211, IC87114) were used (Figure 4A-D). As observed with wortmannin, these inhibitors significantly reversed the cisplatin-induced splicing changes, while no effect was observed in the absence of cisplatin. These results suggest that the class I PI3Ks are involved in the cisplatin-mediated response. RNA-Seq data indicate that p110α and p110β, but not p110γ and p110δ, are expressed in MCF7 cells. P110α, but not p110β, is activated by insulin. At the concentration used, TGX221 and IC87114 did not reduce the phosphorylation of Akt induced by insulin (not shown). Cisplatin treatment did not induce Akt phosphorylation on Ser473 under our experimental conditions (not shown). Moreover, the Akt inhibitors triciribine and MK2206, while efficiently reducing the insulin-induced phosphorylation of Akt, did not affect the cisplatin-induced changes in splicing in AMZ2 and HNRNPDL-E6 (Figure 4E-H). Finally, insulin did not induce a change in AS that was similar to cisplatin in conditions that increase Akt phosphorylation (not shown). Together, these observations strongly suggest that the splicing alterations elicited by cisplatin require p110β, but are independent of Akt.

### Cisplatin-induced alteration of splicing involves SRSF4

**Figure 5:**
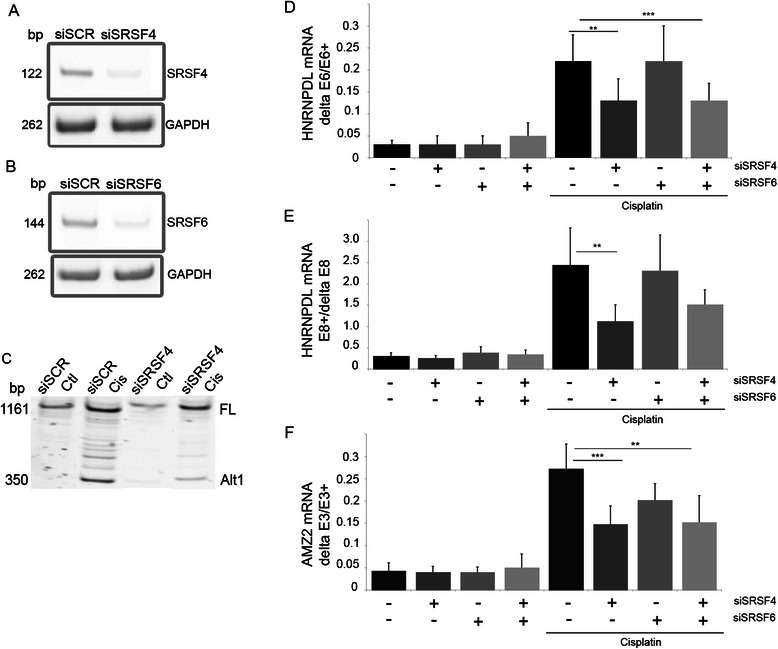
**SRSF4 is involved in cisplatin-induced splicing. A**-**B**: MCF7 cells were transfected with siRNA targeting SRSF4 or/and SRSF6. Cells were harvested three days post-transfection and SRSF4 and SRSF6 mRNA levels were measured by end-point RT-PCR to control the efficiency of the siRNA. **C**-**F**: the histograms and errors bars represent mean and SD, respectively, illustrating the inter-experiment differences in the percentage of exon inclusion (n = 5 to 7). However, the statistics were made on the fold change measured in each independent experiment. MCF7 cells transfected with control siRNA (siSCR) or siRNA targeting SRSF4, SRSF6 or both were treated with cisplatin. The splicing of MDM2 **(C)**, HNRNPDL-E6 (**p = 0.012; ***p = 0.0013) **(D)**, HNRPDL-E8 (**p = 0.0163) **(E)** and AMZ2 (***p = 0.0002, **p = 0.001) **(F)** was evaluated by end-point RT-PCR.

Using a siRNA screen targeting 57 splicing factors, we identified SRSF4 as a regulator of hnRNPDL exon 6 splicing in MCF7 cells in basal growth conditions. We evaluated the role of SRSF4 in mediating the effect of cisplatin on AS by using a siRNA targeting SRSF4 and a control siRNA targeting the splicing factor SRSF6. Reduced levels of SRSF4 mRNA (81 ± 7% reduction, n = 3) and SRSF6 mRNA (80 ± 10%, n = 3) were confirmed by end-point RT-PCR (Figure 5A-B). siSRSF4 alone or when combined to siSRSF6 (siSRSF4/6) partly abrogated the splicing changes induced by cisplatin in the events tested (Figure 5C-F). The control siSCR and siSRSF6 alone had no effect. A similar reduction of the cisplatin-induced HNRNPDL exon 6 exclusion and exon 8 inclusion after knock-down of SRSF4 was observed in the breast cancer cell line BT549 (not illustrated).

### Cisplatin-induced cell death involves SRSF4

**Figure 6:**
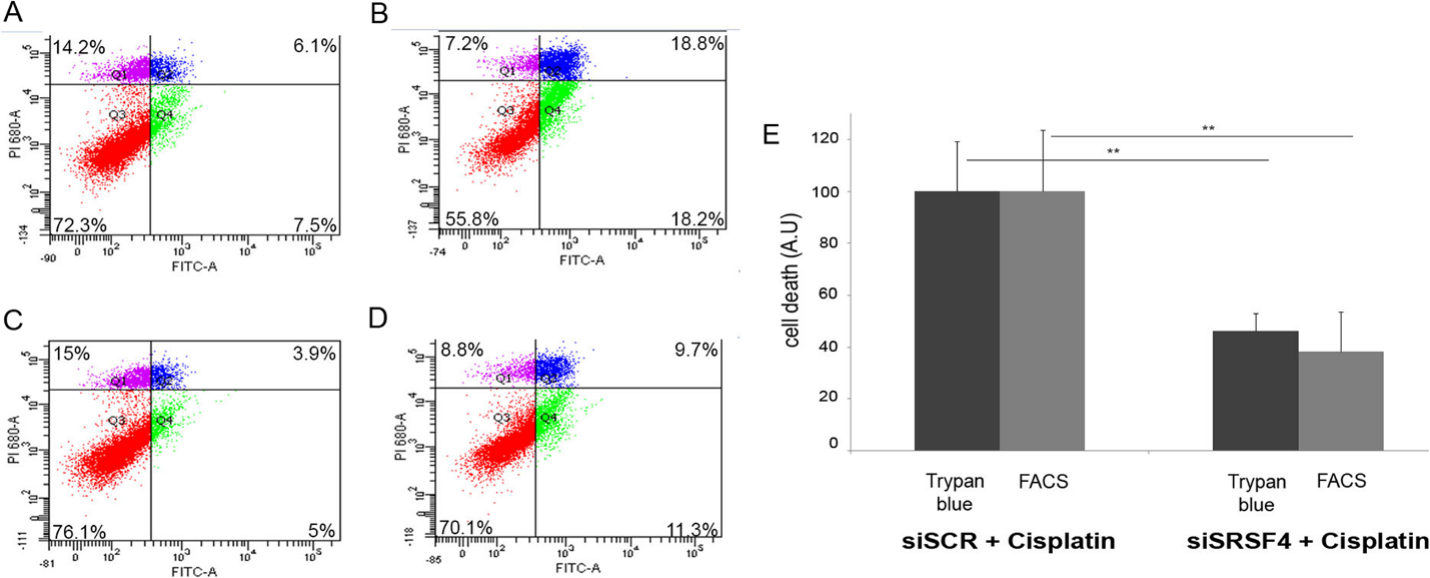
**SRSF4 contributes to cisplatin-induced cell death. A**-**D**: Apoptosis was measured by FACS after annexin V/propidium iodide staining of siSCR **(A,B)** or siSRSF4 **(C,D)** transfected MCF-7 cells untreated **(A,C)** or treated with cisplatin (**B**,**D**; 50 μM, 24 hours). The percentages in each quartile are mean values calculated from three independent experiments. **E**. Quantification of data from trypan blue exclusion and FACS analysis. Histograms indicate cell death in siSRSF4 transfected MCF7 cells treated with cisplatin as compared to death in cells transfected with control siRNA (siSCR) taken as 100%. Data were corrected for cell death measured in untreated cells. Each bar shows the mean with SD of three independent experiments.

To address the potential involvement of SRSF4-dependent splicing events induced by cisplatin in cell death or cell survival, MCF7 cells transfected with the control siRNA siSCR or with siSRSF4 were treated or not with cisplatin for 48 hours. Apoptosis and necrosis were measured by FACS (Figure 6A-D). Although the downregulation of SRSF4 had no effect on growth when cisplatin was absent, it strongly reduced cell death observed in the presence of cisplatin (15 ± 4% for siSRSF4 versus 6 ± 2% for siSCR; p = 0.02), which represents a 62 ± 9% reduction of cisplatin-induced cell death by SRSF4 repression (Figure 6E). These data were further confirmed by using the trypan blue exclusion assay (53 ± 3% reduction, p ≤ 0.01, n = 3, Figure 6E).

## Discussion

The development of chemotherapeutic agents has enabled tremendous progress in cancer therapy. However, the success of these treatments is offset by the development of drug resistance and by toxic side-effects on healthy cells and tissues. The development of this resistance is encouraged by several processes, including decreased access and increased efflux of the drug from the tumor, altered expression of oncogenes, reduced apoptosis and increased DNA repair [27]. In order to evaluate the role of AS in the efficiency of cisplatin, we performed a transcriptome analysis of breast cancer cell line because platinum-based chemotherapy is used as second and third-line of treatment against resistant metastatic breast cancer [28,29]. Moreover, MCF7 cells are well-characterized notably in terms of their response to chemotherapeutic drugs. Our results indicate that cisplatin affects the expression level (absolute fold change >2) of more than 500 genes and provokes changes in at least 700 splicing events, thereby extending previous observations that chemotherapeutic agents affect AS [6,13,16]. This splicing reprogramming also occurs in other transformed cell lines including the breast cancer cell lines MDA-MB-231 and BT549, the endometrial adenocarcinoma cell line Ishikawa and in primary fibroblasts.

Many of the genes whose expression is altered by cisplatin have functions in cell cycle. Cisplatin-induced changes also affect the expression of tumor suppressor genes, oncogenes and genes involved in determining cell fate (Table 4). Strikingly, the list lacks genes encoding splicing factors, suggesting that the impact on splicing control principally stems from post-transcriptional and/or post-translational events affecting their expression, localization and activity. In contrast, cisplatin affected the AS of many splicing factors. Accordingly, our gene ontology analysis suggests that splicing function may be one of the pathways most affected by cisplatin.

We observed that other chemotherapeutic drugs, namely camptothecin and doxorubicin, induce the same changes in AS as those elicited by cisplatin (unpublished work). As these drugs all induce DNA damage, it is tempting to speculate that activation of the DDR pathway may be involved in promoting these splicing alterations. In contrast to this prediction, the genetic depletion and/or the specific inhibition of p53, ATM, ATR and DNA-PK failed to suppress AS re-programming upon cisplatin treatment. These data contrast with those of Shkreta et al. [30] who observed that the shift in Bcl-x splicing induced by oxaliplatin or cisplatin in HEK-293 cells was abrogated by inhibiting ATM, ATR or p53. However, no significant change in Bcl-x splicing by cisplatin was recorded here by deep sequencing or RT-PCR in MCF7 cells (not illustrated), consistent with the very small shift previously observed in MCF7 cells [30]. These discrepancies may be related to the different cell lines used, which may display different thresholds to elicit the DNA damage response.

Previous reports indicate that the PI3K/Akt axis can affect the AS of many primary transcripts at least in part by activation of SRPK and the phosphorylation of SR proteins [31-33]. We investigated the role of this pathway in the AS changes induced by cisplatin by using a panel of inhibitors. Our results indicate that cisplatin alters AS in a process that requires the PI3K subunit p110β. The link between p110β and the splicing events altered by cisplatin remains unclear but is independent of Akt. An intriguing possibility is that cisplatin affects the nuclear activity of p110β, which in turn may directly affect the activity of splicing factors. A role for p110β is not totally unexpected since there is mounting evidence indicating that nuclear lipids can regulate nuclear functions including splicing [34,35]. While phosphoinositides associate with nuclear membranes, they also co-localize in nuclear speckles [36] and interact with various proteins or ribonucleoprotein complexes including the spliceosome components U2 snRNP, U4/U6 snRNP and SF3A1.

We observed that knocking down SRSF4, but not SRSF6, abrogated the cisplatin-induced changes in splicing. CLIP analysis followed by high-throughput sequencing identified GA rich pentamers with ^G^/_A_AA^A^/_G_A sequence as a consensus motif for the binding of SRSF4 to RNA [37]. Moreover, SRSF4 preferentially binds to exons, with a peak of binding ~50 nucleotides upstream of the 5′ splice site. Sequences matching with these sequences are observed in the exons that were skipped in response to cisplatin. However, that they represent binding sites for SRSF4 remains to be tested.

Although SRSF4 may also have an indirect function, for example by regulating the splicing of other splicing factors, we believe that this scenario is unlikely to explain the rapid changes in the steady state levels of splice variants imposed by cisplatin. Nevertheless, portions of the RS-rich regions of SRSF3 and SRSF7 are truncated due to exon skipping (SRSF7) or alternative termination (SRSF3) in response to cisplatin treatment, thereby possibly affecting the phosphorylation of these proteins and their association with other splicing partners.

A link between altered splicing and the efficacy of cancer treatment is suggested by several findings. In lymphocytes of patients with chronic lymphocytic leukemia, mutations in the gene encoding the splicing factor SF3B1 are more frequent after treatment, suggesting a chemotherapy-driven clonal selection for cells being affected in splicing [38,39]. The efficacy of chemotherapetic agents may act at least in part through reprogrammation of AS Consistent with this view, treatments of human 293 cell line with a panel of chemotherapeutic agents induced splicing shifts that encouraged the production of pro-apoptotic variants of Bcl-x, caspase-9 and survivin [6]. Moreover, altering the ratio of splice variants of caspase-9 reduced the resistance of non-small lung cancer cells to various chemotherapeutical agents [40]. On the contrary, splicing switches toward anti-apoptotic versions, as in the conversion from FAS to sFAS, have also been observed ([6] and personal observation). As high sFAS levels correlate with poor survival in patients with T-cell leukemia and gynecological malignancies [41,42], sFAS may contribute to the acquisition of drug resistance and a chemotherapy designed to revert splicing to FAS may increase treatment efficiency [43].

GO terms related to apoptosis were not highlighted by hierarchization analysis of the transcripts alternatively spliced upon cisplatin. We compared a list of transcripts related to apoptosis (GSEA [44,45]) with the list of transcripts with splicing affected by cisplatin treatment. Twenty-six actors involved in the regulation of apoptosis were common to both lists, as for example BAX, caspase-6, caspase-8 (pro-apoptotic) and MADD, API5 (anti-apoptotic). These examples illustrate that cisplatin-induced alterations of splicing may have both anti- and pro-apoptotic effects, and the net effect cannot be estimated on a theoretical basis.

Here, we observed that knocking down SRSF4 reduced the impact of cisplatin on cell death, suggesting an overall therapeutic benefit associated with the expression of SRSF4. Thus, while the pharmacological alterations of splicing induced by chemotherapic agents may fuel therapeutic efficiency, preventing these alterations by inhibiting SRSF4-regulated splicing may help cells to resist the cisplatin treatment. This situation is likely to be more complex given the large number of splicing regulators, their combinatorial mode of regulation and the diversity of their targets. A growing list of pharmacological agents that can modulate splicing is now emerging, with some demonstrating anti-tumor activity [46-48]. Pladienolide, spliceostatin and herboxidiene modulate the function of the spliceosome by binding to the SF3B core component protein [49,50]. A link between splicing alterations and inhibition of cancer cell proliferation was established [50], supporting the concept of using splicing to improve anti-cancer therapy. Another example is provided by the anti-hypertensive agent amiloride that also affects the level and/or the phosphorylation of splicing factors, alters the splicing of cancer genes in various tumor cell lines and sensitizes chronic myelogenous leukemia cells to imatinib [51]. Similarly, dietary agents possessing anticancer activities as curcumin, resveratrol and epigallocatechin-gallate, have been shown to affect splicing, at least in part through modulation of splicing factors levels [52-55].

## Conclusions

We showed that the reprogramming of splicing induced by cisplatin makes a large contribution to its anti-cancer property, and that its action requires class I PI3K p110β and the splicing factor SRSF4. In this context, our data have two major implications. They suggest that pharmacologically modulating AS can potentially affect the success of chemotherapy. Moreover, they raise the interesting possibility that molecules or conditions (as drugs used for non-tumoral diseases, food components and redox status) that modify AS may influence the response to anti-cancer treatments.

## Additional files

Additional file 1:
**Detailed real-time RT-qPCR procedure, according to the MIQE guidelines.**

Additional file 2:
**Sequences of the primers used for RT-qPCR and RT-PCR analyses.**

Additional file 3:
**Differential gene expression of controls and cisplatin-treated MCF7 cells.**

Additional file 4:
**Post-transcriptional events, from RNA-Seq in control and cisplatin-treated MCF7 cells.**
