# Supplementary material for: Role of the splicing factor SRSF4 in cisplatin-induced modifications of pre-mRNA splicing and apoptosis
Source: BMC Cancer. 2015 Apr 7;15:227. doi: 10.1186/s12885-015-1259-0 (PMC4399393; doi:10.1186/s12885-015-1259-0)
Supplement: Additional file 1: — Detailed real-time RT-qPCR procedure, according to the MIQE guidelines. [file 12885_2015_1259_MOESM1_ESM.pdf]

Additional file 1: Detailed real-time RT-qPCR procedure, according to the MIQE guidelines.

Experimental design

Definition of experimental and control groups	Cells treated with cisplatin (50 µM, 24 hours) or untreated and harvested at the same time
Number within each group	2 or 3
Assay carried out by the core or investigator's laboratory?	Investigator's laboratory
Acknowledgment of authors' contributions	Most experiments were performed by M. Gabriel. Primers and assay designs were made by M. Gabriel and ChA Lambert
Sample description	MCF7 and Ishikawa cells
Volume/mass of sample processed	250.000 cells
If frozen, how and how quickly?	-80°C, directly
If fixed, with what and how quickly?	No fixation
Sample storage conditions and duration (especially for FFPE[2] samples)	Stored for several days at -80°C prior RNA purification

Nucleic acid extraction

Procedure and/or instrumentation	As provided by manufacturer
Name of kit and details of any modifications	High Pure RNA isolation kit (Roche, Manheim, Germany)
Source of additional reagents used	None
Details of DNase or RNase treatment	DNAse, as provided by manufacturer
Contamination assessment (DNA or RNA)	None. Primers being systematically located on different exons we consider that potential DNA contaminants are unlikely to produce significant amplification. In addition, only one peak was observed on melting curves
Nucleic acid quantification	Spectrometry
Instrument and method	Nanodrop ND-1000
Purity (A_{260}/A_{280})	1.78<A260/A280<2.11
Yield	19.6 to 240.4 µg/nl
RNA integrity: method/instrument	None
RIN/RQI or C_q of 3' and 5' transcripts	None
Electrophoresis traces	None
Inhibition testing (C_q dilutions, spike, or other)	None.

Reverse transcription

Amount of RNA and reaction volume	1 µg RNA in 20 µl
Priming oligonucleotide (if using GSP) and concentration	anchored oligo-dT
Reverse transcriptase and concentration	Moloney Murine Leukemia Virus RT, 200 U/µl (as provided by manufacturer)
Temperature and time	50°C fir 1 hour, 85°C for 15min
Manufacturer of reagents and catalogue numbers	Superscript III Reverse Transcriptase (Invitrogen, #18080-093)
C_qs with and without reverse transcription	No "No-Reverse Transcrition" control was done. Primers being systematically located on different exons we consider this control less critical than using primers located on same exons.
Storage conditions of cDNA	- 20°C

qPCR target information

Gene symbol	GAPDH: NM_001256799.1; B2M: NM_004048.2; MYB: NM_001161660; BRCA1: NM_007294.3; ACTB: NM_004048.2: RB1: NM_00321.2; JAK2 : NM_004972.3 ; FAS : NR_028036.2 ; SERPINB5 : NM_002639.4.
Location of amplicon	GAPDH: 407-463; B2M: 349-458; MYB: 1444-1611; BRCA1: 5666-5817; ACTB: 359-422: RB1: 2803-2981; JAK2 : 3756-3907 ; FAS : 413-672 ; SERPINB5 : 741-936.
Amplicon length	See additional file 2.

Sequence alignment	BLAST
Secondary structure analysis of amplicon	Not investigated
Location of each primer by exon or intron (if applicable)	Primers were systematicaly chosen on different exons
What splice variants are targeted?	Primers did not dicriminated between the different variants, if any.

qPCR oligonucleotides

Primer sequences	See additional file 2.
RTPrimerDB identification number	NA
Probe sequences	No probe used
Location and identity of any modifications	None
Manufacturer of oligonucleotides	Eurogentec, Seraing, Belgium
Purification method	Desalted
qPCR protocol	50°C for 2min.,95°C for 10min., (95°C for 15sec.,60°C for 1min.)40X, followed by melting curve (from 60°C to 95°C)
Reaction volume and amount of cDNA/DNA	25 µl, amount of cDNA corresponding to 5 ng ARN before reverse-transcription
Primer, (probe), Mg^{2+}, and dNTP concentrations	Primers: 200nM; Mg2+ and dNTP as provided by manufacturer.
Polymerase identity and concentration	as provided by manufacturer
Buffer/kit identity and manufacturer	qPCR MasterMix Plus for SYBR-Green (Eurogentec, Seraing, Belgium)
Exact chemical composition of the buffer	As provided by manufacturer
Additives (SYBR Green I, DMSO, and so forth)	None
Manufacturer of plates/tubes and catalog number	MicroAmp®Fast 96-well Reaction plate (Applied Biosystems by life technologies)
Reaction setup (manual/robotic)	Manual
Manufacturer of qPCR instrument	StepOnePlus (Applied Biosytems)

qPCR validation

Evidence of optimization (from gradients)	None
Specificity (gel, sequence, melt, or digest)	Specificity was ensured by acrylamide gel electrophoresis. Melting curve was systematicaly performed at the end of each amplification.
For SYBR Green I, C_q of the NTC	
Calibration curves with slope and *y* intercept	GAPDH: slope=-3.19, y-int=20.0; B2M: slope=-3.47, y-int=23.5; MYB: slope=-3.65, y-int=31.4; BRCA1: slope=-4.21, y-int=31.2; ACTB: slope=-3.65, y-int=21.0: RB1: slope=-3.84, y-int=22.9; JAK2 : slope=-3.37, y-int=28.2; FAS : slope=-5.03, y-int=29.1; SERPINB5 : slope=-3.61, y-int=30.6.
PCR efficiency (%) calculated from slope	GAPDH: 106; B2M: 94; MYB: 88; BRCA1: 73; ACTB: 87: RB1: 82; JAK2 : 98 ; FAS : 58 ; SERPINB5 : 89
CIs for PCR efficiency or SE	ND
r^2 of calibration curve	GAPDH: 1.00; B2M: 0.98; MYB: 1.00; BRCA1: 0.97; ACTB: 1.00: RB1: 1.00; JAK2 : 1.00 ; FAS : 0.97 ; SERPINB5 : 0.94
Linear dynamic range	From cDNA equivalent to 100 to 0.1ng of RNA before reverse-transcription
C_q variation at LOD	ND
CIs throughout range	ND
Evidence for LOD	ND
If multiplex, efficiency and LOD of each assay	No multiplex

Data analysis

qPCR analysis program (source, version)	StepOne software v2.2.2
Method of C_q determination	Automatic, as provided by manufacturer. We checked that thresholds were crossed during the exponential phase of the amplification reactions.
Justification of number and choice of reference genes	GAPDH, B2M and ACTB were used as calibrators as their expression is not affected by cisplatin treatment as measured by RNASeq analysis.
Description of normalization method	Fold change was calculated by the ΔΔCq (Pfaffl, 2001) using each of the three calibrator genes, and the geometric mean of the fold change

	was calculated
Number and concordance of biological replicates	Triplicate samples
Number and stage (reverse transcription or qPCR) of technical replicates	No technical replicates for RT step. Technical triplicates for qPCR
Reproducibility (interassay variation, CV)	NA. All measurements were done on the same plate for each target gene
Power analysis	$p<0.05$
Statistical methods for results significance	T-test was performed on the mean of the triplicate of all samples between MCF7 controls or treated with cisplatin. (1+ Efficiency)
Software (source, version)	Excel 2007
