# Supplementary material for: Role of the splicing factor SRSF4 in cisplatin-induced modifications of pre-mRNA splicing and apoptosis
Source: BMC Cancer. 2015 Apr 7;15:227. doi: 10.1186/s12885-015-1259-0 (PMC4399393; doi:10.1186/s12885-015-1259-0)
Supplement: Additional file 2: — Sequences of the primers used for RT-qPCR and RT-PCR analyses. [file 12885_2015_1259_MOESM2_ESM.pdf]

Additional file 2: Sequences of the primers used for RT-qPCR and RT-PCR analyses.

RT-qPCR			
Target mRNA	Primers	Cycle number	Lenght (bp)
FAS	Fwd: gttaatgcccaagtgactgacatca<br>Rev: tcatcacacaatctacatcttctgca	40	260
GAPDH	Fwd: cgccccacttgattttgg<br>Rev: atggaaatcccatcaccatctt	40	183
ACTB	Fwd: gccgtgtgaaccatgtgact<br>Rev: gcttacatgtctcgatcccactt	40	213
B2M	Fwd: gagtatgcctgccgtgtg<br>Rev: aatccaaatgcggcatct	40	110
BRCA1	Fwd: ccagatgcctggacagaggacaa<br>Rev: tggctgtgggggatctggggta	40	152
RB1	Fwd: tgaaggatcagatgaagcagatgga<br>Rev: cacagtgtccaccaaggtcctga	40	179
MYB	Fwd: tagaaccccagctatcaaaaggtca<br>Rev: gattcctgtttgatcacatcctgca	40	168
SERPINB5	Fwd: tggaggccacgttctgtatggga<br>Rev: gccatggtgctgggattagtcca	40	196
JAK2	Fwd: ttaccaagaccagatggatgccca<br>Rev: gaaggtcatttctttcatccagcca	40	152

End-point RT-PCR				
Target mRNA	Target exon	Primers	Cycle number	Length (bp)
VEGF	5-8	fwd: cctggtggacatcttccaggagta<br>rev: ctcaccgcctcggcttgtcaca	35	111/121/145/165
MDM-2	E4-11	fwd: acctcacagattccagcttcggaa<br>rev: gctactagaagttgatggctgagaa	34	FL:1161pb/ alt1:±350pb
MTA1	E3-4	fwd: gatccggagaatcgaggagctca<br>Rev: aggaacagctcccgatgccgca	27	FL:295pb/ ΔE4:244pb/ ΔE3:203pb/ ΔE3-4:152pb
SRSF3	E4	fwd: gattatcgtaggaggagtcctcca<br>rev: catttgacctagatcgactacgaga	31	FL:650pb/ ΔE4:179pb
CSDE1	E4	fwd: agcccgcaactctcgcgagaga<br>rev: ccagctgaacgttcccttcgaca	35	FL: 1086pb /ΔE2-4:501pb/ ΔE2-3-4-5:244pb
EIF4A2	E4	fwd: atcgagagcaactggaatgagattg<br>rev: aacatttgttccaccaatgcaggca	31	FL: 354pb/ ΔE4: 207pb
TMPO	E6-E7-E8	fwd: cactcaagcaaagaagagttgagca<br>rev: tgagttctaatggccggcctgca	35	FL: 501pb/ ΔE6: 386pb/ΔE6-7-8:174pb
HNRPDL	E6	fwd: agataccatcaaattggttctgggaa<br>rev: gatgccttgccataagtgctctgt	31	FL: 341pb/ΔE6: 170pb
HNRPDL	E8	fwd: tcaccaaaacaattaccagccatact<br>rev: tgagacataacacagatagcaaagga	31	FL: 338pb/ΔE8:233pb
AMZ2	E3	fwd: gccagtgatctctttggacccatt<br>rev: gtcaaagaggcctgtccaaagaca	30	FL:452pb/ ΔE3:278pb
MAGOH	E3-4	fwd: aagttcggccacgagttcctgga<br>rev: accaaacacttcaggtcctggaca	27	FL:353pb/ΔE3:242pb
NFE2L1	E5	fwd: gcaggacacctgggcaggcga	27	FL:274pb/ΔE5:184pb

		rev: cccggtcagaagaggagacaaga		
STRAP	E2	fwd: agcccgaggcactgcagcagaa<br>rev: catcaattcatctcctgagacagca	27	FL:367pb/ΔE2:232pb
SRSF4	None	fwd: cattctaagagtagatctcggtcca<br>rev: ctcttttccttgctggggctcct	28	122pb
SRSF6	None	fwd: gcgagcgcgtgatcgtagagca<br>rev: attctgtacgaacaggtggtccgt	25	144pb

The number of PCR cycles, the sequence of the primers and the size of the RT-qPCR and RT-PCR products are indicated. The exons targeted by RT-PCR are also mentioned. For RT-qPCR, the PCR conditions for amplification were 15sec. at 95°C and 1min. at 66°C exept for ACTN (15sec. at 95°C; 1min. at 60°C).General information of RT-qPCR analysis are described in additional file 1. For End-point RT-PCR, 10ng of total RNA were reverse transcribed and amplified using GeneAmp Thermostable rTth Reverse Transcriptase RNA PCR kit (Perkin-Helmer, Boston, MA, USA) and specific pairs of primers (Eurogentec, Seraing, Belgium) in an automated thermal cycler (GeneAmp PCR System 2400 or 9600, Perkin-Elmer, Norwalk, CT, USA). The RT step was at 70°C for 15 minutes. Denaturation of RNA/DNA heteroduplexes for 2 minutes incubation at 95°C was followed by PCR amplification for adequate number of cycles (25 to 35) and a final elongation step of 2 minutes at 72°C. The PCR conditions for amplification of the various genes were 15s of denaturation at 94°C; 20s of primer annealing at 66°C and 30s of polymerization at 72°C, exept for MDM-2, CSDE1 E4, TMPO and AMZ2 (15s at 95°C; 20s at 60°C and 60s at 72°C), VEGF-A (20s at 95°C; 30s at 60°C and 60s at 72°C), SRSF4, SRSF6 (15s at 95°C; 20s at 60°C and 10s at 72°C).
