# Supplementary material for: Role of the splicing factor SRSF4 in cisplatin-induced modifications of pre-mRNA splicing and apoptosis
Source: BMC Cancer. 2015 Apr 7;15:227. doi: 10.1186/s12885-015-1259-0 (PMC4399393; doi:10.1186/s12885-015-1259-0)
Supplement: Additional file 3: — Differential gene expression of controls and cisplatin-treated MCF7 cells. [file 12885_2015_1259_MOESM3_ESM.pdf]

Additional file 3. Differential gene expression of controls and cisplatin-treated MCF7 cells.

Gene	Locus	Fold_change	p_value	q_value
-	chr3:152888857-152897509	252.35	2.9E-10	5.1587E-07
SERPINB5	chr18:61144143-61172318	125.81	2.1994E-12	1.5428E-08
POU3F1	chr1:38509522-38512450	104.95	7.6897E-05	0.00615107
NKX1-2	chr10:126133844-126138586	75.41	0.00092482	0.0362893
-	chr13:27840423-27842744	67.52	0.00038061	0.0198025
LAMP3	chr3:182840002-182880667	62.74	0.00046666	0.0228474
-	chr3:152897652-152902938	52.16	4.6066E-08	3.2469E-05
ATF3	chr1:212738675-212794119	51.28	1.4716E-10	3.5796E-07
GADD45A	chr1:68150859-68154021	51.26	3.6262E-11	1.5051E-07
HBEGF	chr5:139712427-139726188	44.24	8.2292E-09	8.3083E-06
HES2	chr1:6472670-6479979	41.20	5.3796E-08	3.5886E-05
NGFR	chr17:47572654-47592382	40.97	2.12E-06	0.00047996
-	chr1:201427965-201431714	39.30	1.236E-05	0.00169751
-	chr10:124944061-124949886	38.20	1.4672E-05	0.00190571
-	chr11:65201932-65203797	33.61	1.0548E-09	1.4593E-06
SNAI1	chr20:48599512-48605420	32.12	1.3471E-05	0.00181665
GPR3	chr1:27719151-27722317	31.57	4.3308E-05	0.00417043
GPR172B	chr17:4935896-4938727	28.94	1.9755E-05	0.00237589
-	chr3:63737820-63741641	28.53	6.7971E-05	0.00562926
-	chr2:91759769-91764245	28.45	2.8348E-05	0.00314237
FLJ26850	chr19:50553936-50570052	28.01	0.00031041	0.0173847
PTAFR	chr1:28473676-28520447	27.93	8.0828E-07	0.00024475
PRODH	chr22:18900286-18924066	26.66	0.00065482	0.0291553
C5orf4	chr5:154198051-154230213	26.66	1.6797E-05	0.00206864
-	chr4:90604622-90609524	22.44	5.4234E-05	0.00489563
PMAIP1	chr18:57567191-57571538	22.18	8.5177E-10	1.2637E-06
HAP1	chr17:39872703-39890898	20.69	0.00071749	0.0308075
FAS	chr10:90694830-90775542	20.59	8.2902E-08	4.9157E-05
GUCA1B	chr6:42151021-42162694	20.36	5.0831E-05	0.00471181
-	chr9:66464189-66465422	19.71	0.00136013	0.0473964
LIF	chr22:30636441-30642796	19.22	9.2333E-08	5.3065E-05
TNFRSF10C	chr8:22941867-22979518	19.07	0.00027382	0.0159326
PDE3A	chr12:20522178-20837041	17.72	7.0721E-13	6.6047E-09
MIR612	chr11:65211298-65212733	17.42	6.0399E-05	0.00521075
KANK3	chr19:8387467-8408146	17.12	0.0004378	0.0219228
DDN	chr12:49388932-49393088	16.88	0.00117524	0.0427898
PDE4C	chr19:18318770-18359010	15.19	0.00030944	0.0173847
BTG2	chr1:203274663-203278729	15.06	1.568E-10	3.5796E-07
CDKN1A	chr6:36644236-36680969	15.06	5.2856E-10	8.9749E-07
-	chr9:139011220-139016125	14.86	0.00022824	0.0139877
GLIPR2	chr9:36136741-36163903	14.81	0.00071923	0.0308469
FDXR	chr17:72858618-72869156	14.61	5.737E-08	3.695E-05
-	chr13:20530047-20532560	14.54	0.00017712	0.0116901
ARC	chr8:143692409-143695833	14.44	1.484E-05	0.00191815
ZNF488	chr10:48355088-48373866	14.39	0.00022657	0.0139208
-	chr9:96197758-96201089	14.22	0.00111627	0.0414094
-	chr11:65203864-65205748	14.13	4.3278E-06	0.00078863
-	chr17:46202261-46205916	13.96	0.00050333	0.0239215
CSF1	chr1:110453232-110473616	13.93	0.0006025	0.0273473
RN5-8S1	chr22:155996-156152	13.54	1.5751E-06	0.00039866
PLK3	chr1:45266035-45272957	12.20	6.4627E-05	0.00543975
SERTAD1	chr19:40928408-40931932	12.11	8.9516E-07	0.00025922
GDF15	chr19:18496967-18499986	11.81	8.7956E-10	1.2637E-06
-	chr14:77561767-77564132	11.66	0.00131723	0.046429
GGT6	chr17:4459536-4463876	11.34	0.00081615	0.0332839
TAP1	chr6:32808493-32846950	11.27	1.1158E-05	0.00158489
BBC3	chr19:47724078-47736023	10.96	0.00022841	0.0139877
SLC35E4	chr22:31031792-31043862	10.77	0.00079094	0.0326839
EFNB1	chrX:68048839-68062006	10.72	2.4259E-06	0.00053307
ISG15	chr1:948846-949919	10.66	2.5459E-05	0.00288198
SESN1	chr6:109307639-109415708	10.61	0.00001186	0.0016409
MAFB	chr20:39314516-39317876	10.50	3.3502E-05	0.00362399
RGAG4	chrX:71130937-71363424	10.46	7.9532E-06	0.00121763

SP6	chr17:45922152-45933240	10.41	0.00109528	0.0408337
ABHD4	chr14:23067146-23081853	10.16	8.6313E-07	0.0002519
-	chr7:76744574-76750792	10.15	0.00017145	0.0114165
SERPINE1	chr7:100770378-100782547	10.13	0.00020411	0.0130338
-	chr11:65210261-65211224	10.05	0.00067456	0.0296805
PROCR	chr20:33759773-33765165	9.99	7.8173E-05	0.00622649
SFN	chr1:27189566-27216869	9.72	5.866E-06	0.00097761
PRICKLE2	chr3:64053639-64211131	9.47	0.00035994	0.0191397
TP53INP1	chr8:95918344-95961615	9.34	2.1124E-07	9.6231E-05
-	chr9:66454654-66457900	9.31	2.0466E-05	0.0024271
EDA2R	chrX:65581617-65859140	9.10	0.00134047	0.0470927
SLC6A8	chrX:152953751-152962048	8.93	0.00011945	0.00873188
LCAT,SLC12A4	chr16:67973786-68002597	8.92	0.00059409	0.0271307
FOS	chr14:75745480-75748937	8.36	4.426E-05	0.00422669
HIC2	chr22:21771692-21805750	8.27	0.00076054	0.0319221
NRG4	chr15:76228349-76304785	8.09	4.4525E-06	0.00079965
DUSP5	chr10:112257624-112271302	7.80	0.00057507	0.026391
RETSAT	chr2:85569077-85581821	7.64	5.353E-05	0.00486538
LACC1	chr13:44410488-44468070	7.54	0.00019253	0.0124915
SESN2	chr1:28585962-28609002	7.51	5.6104E-06	0.00094833
PDE4A	chr19:10527448-10580307	7.51	0.00135248	0.047218
RNF19B	chr1:33402049-33430396	7.47	0.00031717	0.0176837
PLXNB3	chrX:153029635-153044801	7.24	0.00025807	0.0152778
TSPYL2	chrX:53111541-53117728	7.23	4.9245E-05	0.00461764
SAT1	chrX:23801274-23804327	7.09	0.00016551	0.0110603
TUBB2A,TUBB2B	chr6:3153901-3227968	6.50	0.00019133	0.0124736
C1orf63	chr1:25568739-25573985	6.29	0.00072222	0.0308689
PPP1R15A	chr19:49375648-49379319	6.29	6.6693E-05	0.00553642
IER5	chr1:181057637-181062546	6.21	5.9301E-05	0.00513981
MAFG	chr17:79876144-79885587	6.13	0.000325	0.0179927
TP53INP2	chr20:33292147-33301240	5.90	0.00055671	0.025834
ALDH1A3	chr15:101420008-101456860	5.43	0.00026328	0.0155126
PRIC285	chr20:62189438-62205592	5.33	0.00023029	0.0140579
ID2	chr2:8819399-8824656	5.31	0.00089853	0.0354814
TRIAP1	chr12:120881763-120884215	5.17	3.9512E-05	0.00397993
RPS17	chr15:82821160-82824865	5.14	4.172E-05	0.00410126
GPR84,ZNF385A	chr12:54756228-54785107	5.10	0.00014746	0.010197
IRF1	chr5:131817300-131826465	4.88	0.00105245	0.0398735
EPHA2	chr1:16450831-16482582	4.84	0.00021545	0.0134137
ISCU	chr12:108956293-108963160	4.76	0.00020814	0.0131538
C12orf5	chr12:4430358-4469190	4.74	0.00019483	0.0126135
HOXC13	chr12:54332575-54340328	4.68	0.00047788	0.0230965
SLC30A1	chr1:211744873-211752525	4.54	0.00105106	0.0398611
JUN	chr1:59246462-59249785	4.47	0.00063267	0.0283509
PRKAB1	chr12:120105760-120119429	4.42	0.00029523	0.0168635
IRGQ	chr19:44088518-44100287	4.16	0.00041998	0.0211437
HEXIM1	chr17:43224683-43229468	4.06	0.00047402	0.0230225
RAP2B	chr3:152878530-152888759	3.87	0.00139061	0.0479222
USP32	chr17:58253735-58469586	3.66	0.00098603	0.0378386
CBX5	chr12:54624730-54673915	3.82	0.00031027	0.0173847
MTHFD1	chr14:64854758-64926725	3.84	0.00041888	0.0211168
NUCKS1	chr1:205681946-205719382	3.86	0.00056112	0.0259741
PACS1	chr11:65837823-66015985	3.87	0.00066563	0.0294736
NPNT	chr4:106816596-106892828	3.94	0.00136203	0.0474185
UGDH	chr4:39500374-39529218	3.94	0.00054927	0.0256482
KCTD3	chr1:215740734-215795149	3.99	0.00061498	0.0277788
KPNA3	chr13:50273442-50367057	4.03	0.00072505	0.0309543
SLC7A2	chr8:17354596-17428087	4.03	0.00030193	0.0171152
CLIC4	chr1:25071759-25170815	4.05	0.00082342	0.0334344
SORT1	chr1:109852187-109940563	4.07	0.00086495	0.0345941
KIAA1244	chr6:138483052-138665800	4.08	0.00024943	0.014957
BIRC6	chr2:32582095-32843965	4.09	0.00105686	0.0399858
IGF1R	chr15:99192122-99638420	4.09	0.00071445	0.0307476
TACC1	chr8:38585703-38710546	4.11	0.00106737	0.0402755
TMEM48	chr1:54231133-54304225	4.19	0.00146189	0.0491986
SLC7A1	chr13:30083550-30169825	4.20	0.00043847	0.0219272
EMP2	chr16:10622278-10674539	4.20	0.00018075	0.0119085
MAP4	chr3:47892179-48130769	4.21	0.00069822	0.0302582

RB1	chr13:48877882-49056026	4.22	0.00087959	0.0349925
MCM6	chr2:136597195-136634018	4.23	0.00087338	0.0348196
APP	chr21:27252860-27543446	4.25	0.00087138	0.0347913
TPD52	chr8:80947104-81083836	4.25	0.00052038	0.0245445
ABHD2	chr15:89631380-89745591	4.30	0.00048959	0.0234777
BMPR2	chr2:203241049-203432474	4.31	0.00085931	0.0344423
RNF130	chr5:179382473-179499109	4.32	0.00140822	0.0481974
CDC6	chr17:38444145-38459413	4.34	0.00110711	0.0411516
ARHGAP35	chr19:47364302-47508334	4.40	0.00026373	0.0155149
HPS3	chr3:148847370-148939832	4.42	0.00090553	0.0356826
DEK	chr6:18224399-18264799	4.49	0.00014021	0.00988931
XBP1	chr22:29190547-29196560	4.50	5.31E-05	0.00484984
CLSPN	chr1:36197712-36235551	4.50	0.0014804	0.0496426
UCK2	chr1:165796731-165880855	4.52	0.00142071	0.0482849
TPD52L1	chr6:125474878-125584644	4.55	0.00092706	0.0363011
DAAM1	chr14:59655387-59838119	4.56	0.00141947	0.0482849
DNMT1	chr19:10244021-10305755	4.58	0.00068233	0.0298819
SKIV2L2	chr5:54603575-54830873	4.60	0.00141528	0.0482824
BLM	chr15:91260578-91359205	4.60	0.00148685	0.0498142
ZFP36L2	chr2:43449540-43453745	4.61	9.3023E-05	0.00713547
DMXL2	chr15:51739920-51915013	4.62	0.00052691	0.0247278
ERGIC1	chr5:172261222-172379688	4.63	0.0001107	0.00825974
KITLG	chr12:88886569-88974250	4.66	0.00043478	0.0218008
PDE8A	chr15:85523743-85682402	4.68	0.00089483	0.0354104
ZNRF1	chr16:75032914-75144892	4.69	0.00049103	0.0235165
ZFHX3	chr16:72698797-73092534	4.69	0.00134552	0.0470927
SKAP2	chr7:26706687-26904341	4.70	0.00083745	0.0337838
MYO1B	chr2:192110027-192290115	4.73	0.00033166	0.0182466
FANCD2	chr3:10068076-10149915	4.74	0.00132904	0.0467931
SEPT9	chr17:75277491-75561103	4.74	0.00029114	0.0167062
DNAJC1	chr10:22045476-22292650	4.77	0.00107909	0.0405946
LRRC1	chr6:53659777-53788919	4.84	0.00058339	0.0267074
ATP11C	chrX:138808504-139014338	4.90	0.00036019	0.0191397
ZHX3	chr20:39807088-39928739	4.94	0.00137649	0.0476578
SMC2	chr9:106856540-106905332	5.01	0.00083053	0.0336135
BRIP1	chr17:59756546-59940920	5.01	0.00091891	0.0361334
MBNL1	chr3:151980404-152365946	5.04	0.00061662	0.0277859
TBC1D30	chr12:65174208-65274798	5.06	0.00097588	0.03763
IGSF3	chr1:117116973-117210314	5.10	0.00015361	0.0104712
RDX	chr11:110100165-110167437	5.12	0.0001539	0.0104717
SGOL2	chr2:201390864-201448818	5.19	0.00115944	0.0424566
DOPEY2	chr21:37529123-37666583	5.20	0.00019235	0.0124915
DSCAM-AS1	chr21:41384096-42219039	5.22	0.00073525	0.0312082
EFEMP1	chr2:56093096-56151298	5.22	0.00128842	0.0456645
TOP2A	chr17:38544772-38574202	5.23	1.5203E-05	0.00192511
EEA1	chr12:93166284-93323107	5.23	0.00101103	0.0386176
SMCHD1	chr18:2655885-2805015	5.25	0.00018412	0.0120875
SH3D19	chr4:152041432-152246488	5.26	0.00119317	0.0431338
UTP20	chr12:101673904-101780397	5.27	0.0007746	0.0322586
SCIN	chr7:12610202-12693531	5.29	0.00096209	0.0373207
SMARCC1	chr3:47627123-47823405	5.35	0.00011832	0.00866691
SPTBN1	chr2:54683453-54898583	5.36	0.00028726	0.0165342
MAN2A1	chr5:109025155-109205322	5.47	0.00060208	0.0273473
GMPS	chr3:155588324-155659067	5.48	0.00016288	0.010904
ACACA	chr17:35441919-35766902	5.51	0.00069704	0.0302423
SLC38A1	chr12:46576840-46663208	5.52	1.5091E-05	0.00192121
SND1	chr7:127292201-127847924	5.53	2.3042E-05	0.00262423
C8orf83	chr8:93895757-93978372	5.54	0.00129211	0.0457158
USP37	chr2:219314973-219433084	5.57	0.00135436	0.0472396
ITGB6	chr2:160956218-161056823	5.58	0.00079547	0.0327914
MCM3	chr6:52128811-52149582	5.59	0.00092641	0.0363011
CFDP1	chr16:75327607-75467387	5.59	0.0002033	0.0130042
CHD6	chr20:40030753-40247133	5.60	0.00020828	0.0131538
EPS8	chr12:15773005-15942510	5.66	0.00039585	0.0203797
DLGAP5	chr14:55613507-55658396	5.68	0.00047987	0.0231602
ZNF609	chr15:64752978-64978266	5.68	0.00086329	0.0345651
CBFA2T3	chr16:88941262-89043504	5.73	0.00073455	0.0312082
RAD18	chr3:8918879-9005159	5.74	0.00020846	0.0131538

MYO5B	chr18:47349155-47721451	5.74	0.00097295	0.0375859
NUP210	chr3:13357736-13461809	5.80	7.3176E-06	0.00114856
CDK5RAP2	chr9:123151146-123342437	5.84	0.0014257	0.0482849
RAB27B	chr18:52495707-52562747	5.88	5.3679E-05	0.00486706
AREG	chr4:75310852-75320726	5.89	0.00012419	0.00900814
DOCK7	chr1:62920396-63153974	5.90	0.00094018	0.0367207
MTUS1	chr8:17501302-17658727	5.96	0.00059572	0.027172
NR2F2	chr15:96869156-96883492	5.96	0.00011229	0.0083393
EMB	chr5:49691963-49737234	5.97	0.00014061	0.00988931
KIAA1549	chr7:138516126-138666064	6.01	0.00039912	0.020424
MIS18BP1	chr14:45672392-45722605	6.01	0.00019261	0.0124915
FAM111B	chr11:58874657-58910221	6.03	0.00074858	0.0315326
ATP2B1	chr12:89981825-90105729	6.05	5.5855E-05	0.00495609
ATP9A	chr20:50213313-50384908	6.08	6.4239E-06	0.00102992
NASP	chr1:46049370-46084578	6.09	0.00037917	0.0197548
MPHOSPH9	chr12:123634560-123717676	6.14	0.0009722	0.0375859
BUB1	chr2:111394804-111435684	6.14	0.00012538	0.00907679
RBFOX2	chr22:36134782-36424585	6.17	0.00129232	0.0457158
AREG	chr4:75480628-75490485	6.18	0.00010918	0.00818952
AKAP13	chr15:85923850-86292586	6.18	0.00034279	0.0186276
GALNT7	chr4:174089903-174245118	6.26	6.245E-05	0.00531405
ZMYND8	chr20:45837909-45986033	6.30	0.00046221	0.0227429
ATP8B1	chr18:55297533-55470327	6.31	9.6721E-05	0.00737369
PIK3CB	chr3:138371539-138553620	6.32	0.00041873	0.0211168
HLTF	chr3:148747903-148804341	6.33	8.6594E-05	0.00676735
PRR11	chr17:57232859-57284070	6.34	9.575E-06	0.00141377
FAM72D	chr1:143896451-143913143	6.36	0.00095061	0.0370294
ITPK1	chr14:93403258-93582263	6.36	0.00014188	0.0099435
SLC39A11	chr17:70642084-71088853	6.36	0.00094171	0.0367207
RPS6KA3	chrX:20168028-20284750	6.37	0.00044182	0.0220357
WDHD1	chr14:55405655-55493819	6.38	0.00033535	0.0183786
CRIM1	chr2:36583369-36778278	6.42	0.00056665	0.0261031
SLC39A10	chr2:196521531-196602426	6.42	0.00034357	0.0186276
PARN	chr16:14529556-14724128	6.43	0.00014084	0.00988931
TBL1XR1	chr3:176738541-176915048	6.43	0.00014768	0.010197
GALNT3	chr2:166604312-166650803	6.50	6.9819E-05	0.00574481
MSH2	chr2:47630201-47721087	6.50	1.5086E-05	0.00192121
PRIM1	chr12:57123939-57146146	6.53	0.00119137	0.0431249
HMMR	chr5:162887516-162930123	6.53	0.00109519	0.0408337
VRK1	chr14:97263683-97347951	6.53	0.00125981	0.0449061
TTK	chr6:80714321-80753270	6.54	0.00089957	0.035485
ADK	chr10:75910942-76469238	6.55	3.6352E-05	0.00379341
CACNG4	chr17:64960977-65031569	6.56	1.3833E-05	0.00185209
MBNL2	chr13:97874573-98046374	6.58	0.00067698	0.0296821
IRS1	chr2:227596032-227663506	6.60	2.0948E-05	0.00246567
ANKRD28	chr3:15708743-15901053	6.62	0.00010332	0.00778138
ARHGAP39	chr8:145754562-145911113	6.64	0.00140694	0.0481974
MCF2L	chr13:113623534-113754053	6.66	0.00088351	0.0351111
PHF20	chr20:34359922-34538288	6.67	0.00075828	0.0318631
HERC3	chr4:89513646-89629825	6.72	0.00124466	0.0445317
EHMT1	chr9:140513443-140730578	6.72	0.0011725	0.0427317
BRCA1	chr17:41196311-41277500	6.78	0.00073621	0.0312082
VPS13A	chr9:79791671-80032399	6.79	0.00097941	0.0377184
WWC1	chr5:167719064-167899308	6.79	0.00030289	0.0171286
RTKN2	chr10:63952952-64028466	6.80	0.00078213	0.0324995
GPR160	chr3:169755734-169805264	6.82	0.00035926	0.0191397
UNC13B	chr9:35161988-35405332	6.84	0.0003356	0.0183786
ELOVL5	chr6:53132195-53213977	6.86	0.00015318	0.0104612
CDC42BPA	chr1:227177565-227505826	6.89	0.00035375	0.0189051
PLS1	chr3:142315228-142432505	6.92	0.00014753	0.010197
EPB41L2	chr6:131160487-131384462	6.95	0.00102606	0.0391119
THBS1	chr15:39873279-39889668	7.06	8.2552E-07	0.00024475
RALGPS2	chr1:178694299-178890942	7.06	0.00115475	0.0424159
CCNY	chr10:35535952-35860847	7.07	0.00034523	0.0186907
STK39	chr2:168810529-169104105	7.13	0.00051967	0.0245445
RHOBTB3	chr5:95066849-95132071	7.15	1.4579E-06	0.00038085
PREX1	chr20:47122554-47444481	7.22	3.4471E-07	0.00014004
PTPN13	chr4:87515467-87736328	7.24	0.00050761	0.0240945

MLL3	chr7:151832009-152133090	7.24	5.4797E-05	0.00490275
CCNA2	chr4:122722471-122745088	7.29	3.4139E-05	0.00364755
KIAA1324L	chr7:86506222-86689014	7.32	0.00118075	0.0429485
USP13	chr3:179370932-179507321	7.33	0.00041699	0.0211069
NBAS	chr2:15307031-15701454	7.46	0.00110875	0.0411714
STC2	chr5:172741725-172756506	7.50	1.9476E-07	9.1801E-05
ITGA2	chr5:52285155-52390609	7.52	3.4499E-05	0.00367166
TULP4	chr6:158666238-158932858	7.52	0.00070128	0.0303557
ANO6	chr12:45566816-45834187	7.54	0.00021585	0.0134163
LCLAT1	chr2:30670122-30867091	7.59	0.00047142	0.0229804
KIAA0922	chr4:154387497-154558124	7.60	0.00061629	0.0277859
CDK19	chr6:110931180-111136412	7.65	0.00015558	0.0105668
FAM72A	chr1:206138910-206155074	7.65	0.00067283	0.0296396
SPIRE1	chr18:12446033-12657912	7.73	0.00028718	0.0165342
KIF11	chr10:94352824-94415152	7.74	2.8994E-06	0.00061366
PDS5B	chr13:33160563-33352158	7.75	1.1767E-05	0.00163508
C6orf211	chr6:151773421-151791232	7.78	4.5645E-07	0.00016717
SRGAP1	chr12:64238540-64541922	7.88	0.00033603	0.0183786
KIF13B	chr8:28924794-29120610	7.90	0.00032167	0.0178547
COL4A5	chrX:107683073-107940775	7.91	0.00121628	0.043772
SLC25A13	chr7:95749531-95951459	7.95	1.9988E-05	0.00238557
ARHGAP10	chr4:148653388-148993927	7.99	0.00087543	0.0348642
SESTD1	chr2:179966418-180129350	8.04	0.00089331	0.0354095
NCOA2	chr8:71022009-71316037	8.04	0.00014747	0.010197
ARHGAP18	chr6:129898239-130031370	8.10	0.00030921	0.0173847
AR	chrX:66763873-66950461	8.15	7.1012E-05	0.00583012
KYNU	chr2:143588843-143799885	8.16	4.4466E-05	0.00422669
PIP4K2A	chr10:22823765-23003503	8.23	0.00031007	0.0173847
EPB41	chr1:29213602-29450421	8.26	0.00038963	0.0201033
CERS6	chr2:169312758-169642939	8.34	0.00020616	0.0131198
SLC26A2	chr5:149340299-149366963	8.39	5.2278E-05	0.00480399
EXT1	chr8:118811601-119124058	8.40	0.00119688	0.0431927
SP4	chr7:21467688-21554151	8.43	0.00114272	0.0420152
CIT	chr12:120123408-120315104	8.44	0.00096541	0.0374106
SMARCA2	chr9:2015341-2193623	8.48	6.296E-05	0.00533319
UHRF1	chr19:4909509-4962222	8.49	5.603E-05	0.00495989
CENPF	chr1:214776531-214837914	8.49	1.4301E-05	0.00188766
METTL15	chr11:28129797-28355054	8.52	0.00100612	0.0384693
TEAD1	chr11:12695968-12966284	8.53	3.9378E-05	0.00397993
ASAP2	chr2:9346893-9545812	8.58	3.5427E-06	0.00069288
IPO11,LRRC70	chr5:61708572-61924416	8.59	0.00143944	0.0486183
MED13L	chr12:116391709-116715023	8.59	6.3186E-06	0.00101741
PRIM2	chr6:57182421-57513376	8.62	4.7205E-05	0.00447557
RPS6KA5	chr14:91337166-91526993	8.72	0.00098294	0.0377765
MNAT1	chr14:61201458-61435398	8.73	2.9819E-05	0.00329563
PRKAR2B	chr7:106685177-106802256	8.79	0.00144421	0.0486912
STS	chrX:7137471-7272682	8.82	0.00140297	0.0481703
KIF14	chr1:200520624-200589862	8.82	9.1048E-05	0.00702725
SNX10	chr7:26331514-26413949	8.84	0.00037717	0.0197548
SIPA1L2	chr1:232533641-232697222	8.89	6.6407E-05	0.00552701
PDSS2	chr6:107473760-107780779	8.97	0.00024984	0.014957
MBNL3	chrX:131503342-131624046	9.17	0.00035371	0.0189051
SH3PXD2B	chr5:171760502-171881527	9.23	0.00098172	0.0377682
LRBA	chr4:151185624-151936649	9.36	9.7857E-05	0.00744509
TRIO	chr5:14143828-14510146	9.37	0.00070653	0.0305344
FAM117B	chr2:203499900-203634480	9.43	0.00062919	0.0282646
RBM47	chr4:40425271-40632738	9.46	1.4293E-06	0.00037867
FUT9	chr6:96463844-96663488	9.51	4.5584E-06	0.00081108
FNDC3A	chr13:49550047-49783915	9.52	6.5833E-05	0.00550828
TMEM135	chr11:86748885-87039876	9.63	0.00011668	0.00859725
GPR126	chr6:142623055-142767480	9.63	3.3389E-08	2.4457E-05
SLC16A7	chr12:59989888-60179734	9.70	0.0001272	0.00919104
PLEKHA5	chr12:19282625-19529333	9.72	0.00039662	0.0203797
KCNK5	chr6:39156746-39197251	9.76	0.00050939	0.0241279
ADD3	chr10:111705316-111895323	9.79	0.00110654	0.0411516
SH3RF1	chr4:170015406-170192249	9.86	0.00024687	0.0148986
SCFD2	chr4:53739150-54232242	9.87	0.00021456	0.0133811
ASAP1	chr8:131029374-131455906	9.91	2.8642E-06	0.00061141

FAM102B	chr1:109102970-109181949	9.95	2.8255E-08	2.2457E-05
PBX1	chr1:164528595-164823469	9.97	3.9585E-06	0.00075062
RTTN	chr18:67671042-67872962	10.02	0.00057127	0.0262812
RANBP17	chr5:170288895-170727019	10.03	0.00084571	0.0339337
NEO1	chr15:73344824-73597547	10.09	1.0046E-05	0.00146026
PTK2	chr8:141668480-142011412	10.10	5.0701E-05	0.00471136
FAM72B	chr1:120839004-120855681	10.10	0.00048659	0.023364
CXCR7	chr2:237478379-237490994	10.11	3.4488E-07	0.00014004
ALCAM	chr3:105085556-105295757	10.15	1.44E-06	0.00037881
ZNF618	chr9:116638561-116818875	10.23	4.0483E-05	0.00403618
SLC41A2	chr12:105197249-105352049	10.26	0.00108545	0.0406699
IL1R1	chr2:102770401-102796334	10.30	0.00012029	0.00877614
C1orf21	chr1:184356149-184598155	10.33	6.5897E-06	0.00105198
FAF1	chr1:50905240-51425936	10.34	2.1733E-05	0.0025213
-	chr12:60179942-60183516	10.37	0.00040198	0.0205139
SRGAP2P2	chr1:143914207-144094477	10.38	0.00047185	0.0229804
WWC2	chr4:184020462-184241929	10.46	0.00013254	0.00943076
XPR1	chr1:180601145-180859415	10.55	1.5588E-06	0.00039866
NDC80	chr18:2571509-2616634	10.59	0.00031892	0.0177544
CA12	chr15:63615729-63674075	10.60	2.2603E-05	0.00260603
ZNRF3	chr22:29279754-29453476	10.62	0.00048218	0.0232417
KIAA0146	chr8:48173488-48648563	10.67	0.00081861	0.0333189
ZSWIM5	chr1:45482075-45672250	10.86	0.0007869	0.0325759
ARL13B	chr3:93698982-93774522	10.89	0.00071574	0.0307678
ZHX2	chr8:123792497-124014800	11.02	0.00018465	0.0121015
RASSF8	chr12:26107587-26232825	11.10	4.881E-06	0.00084806
EXOC4	chr7:132937822-133750579	11.18	0.00019944	0.0128487
JAK2	chr9:4985244-5128183	11.31	5.4518E-05	0.00489563
SASH1	chr6:148663728-148873184	11.31	0.00011723	0.00860338
CABLES1	chr18:20714527-20840434	11.36	5.8655E-05	0.00510751
VAT1L	chr16:77822482-78014001	11.46	0.00027197	0.0158992
SFMBT2	chr10:7200585-7453448	11.47	0.00026508	0.0155453
SORL1	chr11:121322911-121504471	11.53	7.217E-05	0.00586085
NEBL	chr10:21068902-21463852	11.65	1.546E-06	0.0003983
FREM2	chr13:39261172-39461267	11.69	1.0888E-06	0.00031048
DPYSL2	chr8:26371708-26515693	11.70	0.00025025	0.014957
DEPDC1B	chr5:59892738-59995993	11.73	0.00025184	0.015014
TBC1D5	chr3:17198653-17784240	11.79	1.9296E-05	0.00234028
WDR70	chr5:37379411-37752774	11.80	7.7281E-05	0.0061686
VAV2	chr9:136627015-136857482	11.85	4.1981E-06	0.00077839
CRADD	chr12:94071150-94244531	11.85	0.00078349	0.0325201
C6orf97	chr6:151815174-151942451	11.87	1.3756E-05	0.0018484
NFIB	chr9:14081841-14398982	11.98	0.0002097	0.0131796
PRKCA	chr17:64298925-64806862	12.49	7.9776E-05	0.00631433
ADAMTS19	chr5:128795974-129074588	12.50	0.00030078	0.0171152
CTDSPL	chr3:37903668-38025960	12.56	0.00032981	0.0181986
SIPA1L3	chr19:38397867-38699008	12.57	4.3316E-05	0.00417043
FGD4	chr12:32655040-32798984	12.62	0.00021864	0.0135223
PMP22	chr17:15133095-15168644	12.64	5.234E-05	0.00480399
ANK3	chr10:61786055-62493284	12.65	0.00013826	0.00980055
CEP128	chr14:80962820-81408057	12.66	0.00096115	0.0373207
SYTL5	chrX:37765066-37988073	12.66	1.0405E-05	0.00150074
TBC1D4	chr13:75858808-76056267	12.76	4.8244E-05	0.00456258
DDAH1	chr1:85784167-86044046	12.83	4.4018E-06	0.00079436
GULP1	chr2:189156395-189460652	12.87	7.1632E-05	0.00584523
HIPK2	chr7:139246315-139477693	12.93	3.8004E-05	0.00388594
GAS2L3	chr12:100967465-101022041	12.95	0.00143847	0.0486183
DCDC2	chr6:24171982-24383520	13.08	4.1516E-06	0.00077839
MYO10	chr5:16662015-16936385	13.12	4.7482E-06	0.00083216
STK3	chr8:99466858-99954799	13.23	3.1592E-05	0.00347102
AIM1	chr6:106808687-107018334	13.29	9.6563E-06	0.00141459
NCOA1	chr2:24714421-24993570	13.34	8.4776E-06	0.00127697
SDC2	chr8:97505881-97624037	13.34	0.00084325	0.0339181
WIPF1	chr2:175424301-175547627	13.34	0.0001466	0.010197
MPP7	chr10:28339922-28591846	13.35	1.4121E-05	0.00187719
BEND3	chr6:107386384-107435636	13.46	0.00013682	0.00971676
CAMK2D	chr4:114372187-114683083	13.61	0.00136402	0.0474435
PRKCH	chr14:61788514-62017698	13.67	0.00103273	0.039286

FNDC3B	chr3:171757417-172118492	13.76	0.0013925	0.047943
GREB1	chr2:11622955-11782964	13.77	9.2378E-06	0.00138035
THADA	chr2:43457974-43823185	14.05	0.00046816	0.0228909
ESR1	chr6:152011630-152424580	14.28	2.3032E-05	0.00262423
PDZRN3	chr3:73431651-73674072	14.29	0.00079748	0.0327914
NFATC2	chr20:50003503-50179168	14.32	0.00104638	0.039724
TRPS1	chr8:116420723-116681328	14.42	1.2059E-07	6.1707E-05
FUT8	chr14:65877309-66210839	14.53	2.042E-07	9.4174E-05
NPAS2	chr2:101436612-101613287	14.54	0.00079749	0.0327914
LMNB1	chr5:126112314-126172712	14.56	4.0696E-08	2.9235E-05
AFAP1L2	chr10:116054582-116164515	14.60	0.00073685	0.0312082
-	chr12:131717235-131719769	14.75	0.0010297	0.0392106
GNAQ	chr9:80331021-80646219	14.89	4.0871E-05	0.0040498
ITPR2	chr12:26488284-26986131	14.99	5.9216E-06	0.0009788
TNS3	chr7:47314751-47621742	15.03	1.7549E-06	0.00042875
EML1	chr14:100259744-100408395	15.22	4.3535E-05	0.00418075
TRERF1	chr6:42192668-42419872	15.42	1.512E-05	0.00192121
RALGAPA2	chr20:20370207-20693266	15.68	5.6629E-07	0.00019406
MME	chr3:154742007-154901529	15.73	0.0007312	0.0311457
WDR7	chr18:54318615-54697036	15.89	0.00018322	0.0120497
PTPRK	chr6:128289923-128841870	16.19	1.9673E-09	2.4497E-06
FBXL17	chr5:107194733-107717799	16.23	0.00022038	0.0136072
SVIL	chr10:29745950-30024737	16.31	1.4671E-05	0.00190571
TBC1D22A	chr22:47158517-47571342	16.59	7.1665E-05	0.00584523
RNGTT	chr6:89319988-89673348	16.75	3.5783E-06	0.0006962
MAGI3	chr1:113933149-114230519	16.78	9.0554E-05	0.00700358
XXYLT1	chr3:194788676-194991895	16.89	0.00038627	0.0200129
JPH1	chr8:75146938-75233670	17.47	3.4873E-06	0.00068691
CAMKMT	chr2:44589042-44999729	17.48	0.00111835	0.0414453
TMEM164	chrX:109245862-109421016	17.49	8.018E-05	0.00631433
SRGAP2	chr1:206516199-206637783	17.58	9.3602E-05	0.00716515
TBL1X	chrX:9431334-9687780	17.69	2.5126E-06	0.0005457
PARD3	chr10:34398487-35104253	17.86	3.396E-05	0.00364755
PSD3	chr8:18384812-18871196	18.00	2.5657E-08	2.0836E-05
FAM172A	chr5:92953430-93447404	18.02	4.3575E-06	0.00079018
WDR19	chr4:39184023-39287430	18.03	0.0006202	0.0279133
JARID2	chr6:15245805-15522253	18.03	4.9134E-06	0.00084975
STAG1	chr3:136054668-136471245	18.37	5.5346E-07	0.00019335
PPM1H	chr12:63037762-63328665	18.46	2.3948E-07	0.00010778
-	chr10:9012174-9016319	18.65	0.00065417	0.0291553
PPP3CA	chr4:101944586-102268628	18.66	5.8699E-11	2.1275E-07
JAKMIP2	chr5:146939556-147162268	18.71	2.3808E-06	0.00052939
THSD4	chr15:71407946-72075722	18.80	3.5747E-05	0.00376519
MPP6	chr7:24613084-24729244	18.81	0.00047245	0.0229804
IGFBP5	chr2:217536827-217560272	19.24	2.478E-12	1.5428E-08
DEPTOR	chr8:120885899-121063157	19.60	0.00015839	0.0107189
LRRC20	chr10:72058728-72142382	19.66	4.2456E-05	0.0041517
KIFAP3	chr1:169890466-170043879	20.03	7.9572E-05	0.00631433
NECAB1	chr8:91803920-91997485	20.12	3.4938E-06	0.00068691
NCAM2	chr21:22370632-22912741	20.19	1.6493E-06	0.00041349
CSGALNACT1	chr8:19261311-19540261	20.25	0.00041514	0.0210421
EEFSEC	chr3:127872312-128127489	20.38	6.1072E-05	0.00524457
PGR	chr11:100900354-101000544	20.41	3.7422E-06	0.00072432
MYB	chr6:135502452-135540311	20.44	0.00021074	0.0131997
CMIP	chr16:81478774-81745367	20.59	4.1089E-05	0.00404989
ARID1B	chr6:157099063-157531913	20.66	2.0034E-06	0.00047351
PPM1L	chr3:160473995-160793360	20.69	2.2788E-05	0.00261128
IKZF2	chr2:213864410-214016333	20.70	7.4816E-05	0.00604944
GFRA1	chr10:117816435-118033126	20.72	2.53E-10	4.7256E-07
MAP2K5	chr15:67835020-68099455	20.75	0.00012905	0.00928889
HLCS	chr21:38123188-38362545	20.80	0.00137784	0.0476578
BUB1B,PAK6	chr15:40453209-40569688	21.07	1.6632E-05	0.00206864
-	chr13:23489827-23492386	21.25	9.5952E-05	0.00732999
DDX10	chr11:108535815-108811648	21.26	1.7587E-06	0.00042875
EYA2	chr20:45523262-45817492	21.27	0.00060972	0.0276081
ATP8A1	chr4:42410391-42659122	21.88	0.00020974	0.0131796
RAB30	chr11:82688389-82782884	21.98	4.2591E-05	0.0041517
CDCA7L	chr7:21582832-21985542	22.06	0.00066591	0.0294736

BBX	chr3:107241782-107530049	22.19	1.1936E-07	6.1707E-05
CAB39L	chr13:49882785-50018221	22.56	0.00024502	0.0148106
PEAK1	chr15:77400497-77712446	22.67	3.1909E-06	0.00065136
EXOC6	chr10:94594469-94819251	22.79	1.6606E-06	0.00041355
MAN1A1	chr6:119498385-119670926	22.90	3.83E-06	0.00073565
STARD13	chr13:33677271-34250932	23.11	3.5882E-05	0.00376519
GPC6	chr13:93879077-95060273	23.19	1.3272E-07	6.69E-05
RPTOR	chr17:78518624-78940173	23.38	2.4116E-06	0.00053307
ST8SIA6	chr10:17357417-17497584	23.69	1.0872E-06	0.00031048
ADAM22	chr7:87563565-87832204	23.86	0.0011646	0.0425684
PLCB1	chr20:8113295-8865547	24.00	4.3044E-07	0.0001608
DOCK9	chr13:99445740-99738660	24.13	0.00040367	0.0205722
SGCG	chr13:23733218-23899304	24.16	0.00033685	0.0183967
FGF12	chr3:191857181-192445388	24.42	5.8031E-06	0.00097649
UBE2E2	chr3:23244649-23648882	24.82	9.8519E-06	0.0014376
FIGN	chr2:164464117-164592513	25.11	6.5155E-05	0.00546951
PLCB4	chr20:9049700-9461462	25.23	1.3374E-06	0.00036203
ZNF704	chr8:81540685-81787016	25.56	1.7163E-06	0.00042459
FGF13	chrX:137713733-138287185	25.68	0.0001016	0.00768323
FTO	chr16:53737874-54148379	25.86	5.3379E-07	0.00018991
CAMK1D	chr10:12391582-12877545	26.43	0.00128795	0.0456645
GRB10	chr7:50657759-50861159	26.64	0.00027371	0.0159326
CCDC91	chr12:28343321-28703099	27.17	7.5725E-05	0.00608339
CDKAL1	chr6:20534687-21232634	27.23	2.2789E-06	0.00051283
HSD17B11	chr4:88257690-88312455	27.38	6.0787E-06	0.00098302
SCAPER	chr15:76640526-77176217	27.49	9.2474E-05	0.00710791
TMTC2	chr12:83080933-83528067	27.52	2.0942E-06	0.00047993
SATB2	chr2:200134222-200337481	27.61	0.00025022	0.014957
GPC5	chr13:92050934-93519487	27.88	0.00073095	0.0311457
MGAT5	chr2:135010710-135212192	27.88	5.695E-08	3.695E-05
ERC1	chr12:1099718-1605100	28.07	2.9387E-07	0.00012618
ZMAT4	chr8:40388110-40755343	28.13	0.00107327	0.0404573
ST7	chr7:116592500-116870201	28.44	0.0007953	0.0327914
COL12A1	chr6:75794041-75915895	28.58	4.9891E-08	3.3886E-05
SNX29	chr16:12070601-12668146	28.73	1.3936E-06	0.00037184
LRRC16A	chr6:25279655-25620758	28.89	1.1629E-07	6.1182E-05
MID1	chrX:10413349-10851809	28.93	6.5912E-05	0.00550828
GREB1L	chr18:18822202-19105372	29.14	6.9643E-05	0.00574297
LDLRAD3	chr11:35965611-36253632	29.94	0.00061154	0.0276568
LARGE	chr22:33669061-34316416	29.98	3.0086E-07	0.00012772
NBEA	chr13:35516423-36705514	30.53	3.3048E-06	0.00066732
SIPA1L1	chr14:71787185-72207701	30.87	2.0962E-08	1.9099E-05
VAV3	chr1:108113781-108507545	31.24	0.00023897	0.0144682
MAP3K5	chr6:136878186-137113656	31.41	0.00017506	0.011595
REEP1	chr2:86441119-86565206	32.46	0.00095467	0.0371486
-	chr11:82684281-82686552	33.99	7.6276E-05	0.00611454
TP63	chr3:189349215-189615068	35.14	0.00113774	0.0419147
XRCC4	chr5:82373316-82649579	35.30	6.2808E-05	0.00533239
-	chr5:67096878-67104649	35.35	5.8167E-05	0.00508871
SLC6A14	chrX:115567746-115592625	35.64	1.9611E-10	3.8558E-07
PRLR	chr5:35048860-35230823	36.07	0.0006017	0.0273473
SYT1	chr12:79257772-79845788	36.11	3.8598E-06	0.00073565
ELOVL6	chr4:110967047-111119820	36.47	1.6834E-05	0.00206864
APBB2	chr4:40812043-41216635	37.13	3.8451E-09	4.3434E-06
EFNA5	chr5:106712589-107006596	38.41	4.325E-06	0.00078863
GJA1	chr6:121756744-121770873	38.88	1.7734E-08	1.6561E-05
UST	chr6:149068270-149398126	41.05	0.00136879	0.0475651
BMPR1B	chr4:95679127-96079601	41.15	4E-07	0.00015247
C18orf1	chr18:13217538-13652753	44.12	8.965E-08	5.2328E-05
ROBO1	chr3:78646387-79817059	45.53	1.9414E-09	2.4497E-06
NEGR1	chr1:71868624-72748277	48.24	4.2091E-06	0.00077839
EYA4	chr6:133562479-133853258	48.56	3.8411E-05	0.00389912
CADPS2	chr7:121958477-122526813	49.13	0.00010607	0.00797264
SLCO3A1	chr15:92396937-92715665	49.21	0.00071229	0.0307211
SAMD12	chr8:119201694-119738306	50.12	2.0154E-06	0.00047351
NFIA	chr1:61542945-61928460	52.90	7.5235E-07	0.00023818
LOC100507421	chr7:140774031-141180179	54.68	9.8774E-09	9.7101E-06
SULF1	chr8:70378858-70573147	54.96	3.1147E-06	0.00064284

MAGI1	chr3:65339204-66024509	55.54	1.0443E-07	5.789E-05
HS6ST3	chr13:96743092-97491816	61.53	5.0016E-07	0.00017965
PLCH1	chr3:155197670-155422053	61.75	2.099E-05	0.00246567
PPP1R9A	chr7:94536948-94925727	66.66	8.1845E-06	0.00124284
KCNJ8	chr12:21917888-21927747	67.24	1.2001E-06	0.00033327
-	chr3:160793476-160796882	67.30	1.0653E-05	0.00152475
MLLT3	chr9:20344967-20622514	67.45	0.000252	0.015014
PLXDC2	chr10:20078880-20578372	69.86	8.6254E-07	0.0002519
-	chr5:108530016-108532266	72.26	0.00109723	0.0408654
SEMA5A	chr5:9035137-9546233	75.23	3.7838E-07	0.00015037
ERBB4	chr2:212240441-213403352	80.93	3.8487E-06	0.00073565
LTBP1	chr2:33172368-33624575	94.44	4.9652E-08	3.3886E-05
TIAM1	chr21:32490735-32931290	146.00	0.00011381	0.00841867
GPHN	chr14:66974124-67648525	159.40	6.1825E-08	3.8493E-05

The genomic location of gene, the value for controls and cisplatin-treated MCF7 cells, the fold change, the q-value and p-value were indicated.
