# Supplementary material for: Role of the splicing factor SRSF4 in cisplatin-induced modifications of pre-mRNA splicing and apoptosis
Source: BMC Cancer. 2015 Apr 7;15:227. doi: 10.1186/s12885-015-1259-0 (PMC4399393; doi:10.1186/s12885-015-1259-0)
Supplement: Additional file 4: — Post-transcriptional events, from RNA-Seq in control and cisplatin-treated MCF7 cells. [file 12885_2015_1259_MOESM4_ESM.pdf]

Additional file 4. Post-transcriptional events, from RNA-Seq in control and cisplatin-treated MCF7 cells.

Gene Symbol	RPKM MCF7 Control	RPKM MCF7+ Cisplatin	Alt Splice RPKM	Alt Protein RPKM	Splice Events	Uniprot Events
ABCB6	11	55	0	6	PS-	t (2)
ABCC3	16	16	0	0	PS+	
ABCD4	9	11	2	0	ES- (18.2)	
ACAD8	10	9	0	9	ES+ (2, 3, 4)	
ACIN1	24	29	3	22	ES- (4), ES+ (9.3)	D (1), D (1), D (1), r (1)
ACOT2	8	7	1	0	ES- (2.3)	
ACOT9	11	8	1	8	ES+ (6)	
ACP1	54	52	3	18	ES+ (4.1)	
ACP6	16	7	2	10	AT+ (7)	
ACYP1	7	7	3	0	ES+ (4)	
ADAM15	44	57	1	23	ES+ (20.2), ES- (20.1), ES- (21.1)	D (18), T (18)
ADD1	39	28	2	9	AP- (1)	R (19), R (19)
ADI1	32	39	2	11	AP+ (2)	
ADNP	47	31	2	0	ES+ (2), AP+ (4.1)	
ADPGK	33	38	1	7	AP- (1.1)	
AEBP2	18	10	1	5	AP+ (2.1)	R (6), D (6), R (2.3), D (2.3)
AFMID	5	7	1	0	ES- (10, 11.1)	
AIG1	18	6	2	9	AT+ (3)	
AK2	32	38	1	11	AT- (6)	B (3), R (3), s (3), D (3), R (3)
AKAP17A	8	28	2	11	ES- (4.2, 4.3), AT+ (4.4)	
AKAP9	27	16	3	20	ES+ (28.2)	
AKT1S1	14	31	3	6	ES- (2.3)	
ALDOA	1008	1172	1	0	ES+ (6.2)	
ALG3	23	26	2	15	ES+ (2.2)	t (2.1)
ALG8	49	21	0	11	ES- (14)	t (15), t (15)
ALKBH2	18	25	0	6	ES- (3)	R (4), m (4), R (3), D (4), R (3), R (4), R (3), R (3), m (4), D (4), B (4), D (4), D (3), m (4)
AMD1	51	23	1	26	ES+ (3, 4, 5), ES+ (4, 5)	A (1), D (4), D (4), B (4), S (4), p (1), B (1), A (4), D (4), p (1), A (1), A (4)
AMZ2	80	154	8	40	ES+ (3)	
ANKRD46	5	12	2	7	ES- (3)	
ANP32E	75	29	2	8	ES+ (5.1, 5.2)	
ANXA2	513	478	7	0	ES+ (2)	
AP4B1	18	16	1	3	AP+ (1.1)	
AP4E1	16	16	0	10	PS+	
API5	50	64	2	15	AT+ (14.2)	s (1)
APTX	12	16	4	3	ES+ (10), PS+	D (2), M (7.4), D (5.2), D (5.2), R (2)
AQR	9	6	0	0	ES- (29.2)	
ARFRP1	11	12	0	1	AP+ (3.1)	R (8)
ARHGAP12	26	13	2	13	ES+ (8)	R (5), D (5)
ARHGEF12	21	10	2	11	ES+ (5)	
ARID4B	11	5	1	7	ES- (18)	
ARIH2	13	17	3	0	ES- (5), PS+	
ARL6IP4	48	54	3	27	ES- (3.1)	
ARL8B	39	32	2	12	AT+ (3.3)	R (1.3), i (1.3), R (4), R (6), i (1.3)
ARNT	20	16	3	18	ES+ (7)	
ASCC1	15	7	2	10	ES- (16)	
ASL	13	14	1	4	AP- (2.1)	
ASNS	75	76	2	3	ES- (2.3)	D (4.2), D (4.2)
ATG9A	8	11	1	10	AP- (1)	
ATP1B3	70	64	2	11	ES+ (4, 5, 6, 8)	s (6), D (3), T (3), D (5), D (5), s (5), g (5), s (5), D (6), g (9)
ATP2A3	36	32	1	6	ES- (21.2)	D (20), T (20)
ATP2C1	30	16	1	15	ES+ (29.1)	D (3.3), t (28), T (3.3), R (28), T (29.1), D (29.1)
ATP5C1	249	247	7	103	ES+ (9)	
ATP6AP2	82	149	3	32	ES+ (4, 5.1, 5.3), ES+ (5.1, 5.3)	D (2), D (1), p (1), T (2)
ATP6V0B	40	84	3	19	ES- (7.2)	D (2.2), T (1.1), R (1.1), D (7.1), T (7.1), t (1.1), T (2.2)
ATP6V1D	25	53	2	12	ES+ (7)	
AURKA	70	17	1	2	ES- (3)	D (7), D (7)

B2M	61	116	3	0	AT+ (2.8)	
B3GALNT1	45	14	1	0	ES- (8)	g (9.2), g (9.2), g (9.2), R (9.1), T (9.1), g (9.2)
B4GALT3	7	12	3	0	ES+ (3.5)	
BAG5	32	51	0	13	AP- (2.1)	
BAX	41	119	5	12	ES- (5)	t (6.4), M (6.1), R (6.1)
BCAS1	22	8	1	12	ES+ (11)	
BCL7B	25	27	0	8	PS+	
BLOC1S2	34	131	2	19	AP+ (2.1)	
BRD3	10	8	1	0	AP- (2)	
BRPF3	12	19	0	0	PS-	
BTF3	381	687	7	55	ES- (1.2)	
BTN2A1	6	17	0	7	ES+ (4)	
BUD31	64	81	4	8	ES- (4)	M (3.2)
C11orf48	28	23	0	3	ES- (1.6)	
C11orf57	6	7	2	0	ES- (2.2, 2.3)	
C11orf82	12	17	2	9	ES- (3)	
C12orf10	34	39	2	19	AP+ (1.2)	
C12orf29	9	9	1	0	ES+ (6.1)	
C12orf41	18	43	0	15	AT- (7)	
C14orf101	7	6	1	0	ES+ (12)	
C14orf118	9	18	3	12	AP- (1.1)	
C14orf2	74	106	8	6	ES- (7.2), AT+ (7.2)	
C14orf79	5	16	0	5	ES+ (2.3, 2.4)	
C17orf62	18	24	5	0	ES+ (4), AP- (1)	
C17orf81	12	13	1	10	ES- (7.4), PS-	
C19orf42	13	11	3	6	ES+ (6)	T (8.1), T (2), D (2), D (8.1), t (6)
C5orf24	16	19	1	10	ES+ (4.2)	
C5orf45	9	12	2	6	ES- (2.3), ES- (2.2, 2.3)	
C7orf23	26	28	3	11	AT+ (4.2)	t (5), t (4.1)
C8orf59	31	25	16	9	ES+ (2.1, 2.2, 2.3), ES+ (2.2, 2.3)	
C9orf25	5	10	1	6	ES- (2.2)	
C9orf7	6	8	1	0	ES- (3)	
C9orf80	14	7	1	0	ES+ (2)	
CAPN7	19	20	0	0	ES+ (3), PS+	
CASP6	11	10	1	8	ES+ (4), PS+	
CASP8	5	5	1	0	ES+ (10, 11.1)	
CBS	59	42	1	9	ES+ (5.2)	m (5.2), m (5.2)
CCDC115	15	28	1	35	ES- (1.2)	o (1.2)
CCDC14	9	6	2	0	ES- (2.3)	
CCDC58	26	21	2	19	ES+ (2, 3), ES+ (1.2, 2, 3)	o (3)
CCNL2	23	61	2	14	ES- (9, 11), PS+	R (4), R (1.1), R (4), R (1.1)
CCNT1	13	28	0	0	PS-	
CCT7	320	351	2	74	ES+ (3, 4)	
CD55	15	12	2	12	ES- (10.1)	
CDC25B	49	34	1	23	ES+ (3.1)	
CDK1	54	32	4	21	ES+ (5.1)	D (2), D (2), A (5.1)
CDK10	15	18	2	3	ES- (4)	B (6), D (5), D (6), D (5)
CDK2AP2	38	35	2	6	AT+ (3.2)	
CDK7	29	36	2	5	ES+ (5.1, 6)	D (1), B (2.2), D (1), D (2.2)
CDV3	52	52	2	14	ES+ (7.2)	
CDYL	23	13	2	8	AP+ (5)	D (4)
CENPM	13	7	3	9	AP- (1)	
CENPN	7	20	1	11	ES- (1.2)	
CENPN	36	19	0	5	AT+ (13)	
CERCAM	12	9	0	10	ES+ (3.3)	
CERS5	10	16	0	9	PS+	D (8.2), g (1), T (8.2), N (8.2), t (1), T (15.1), R (1), T (1), R (15.1)
CHCHD7	8	18	2	11	ES+ (2.1, 2.2)	
CHD3	42	49	2	33	ES+ (34)	B (32), R (32)
CHD8	13	15	1	0	AP- (1)	
CHMP1A	58	75	1	9	ES- (5.2)	
CHMP2B	42	41	0	20	ES+ (2)	o (2)
CHTF18	16	13	0	4	PS+	
CHTF8	46	86	3	5	ES- (4)	
CHURC1	33	38	4	16	ES+ (3.1, 3.2), ES+ (3.2)	m (3.2), m (3.2), R (5), R (5), m (3.2), R (3.2), R (3.2), m (5), R (3.2), m (3.2), m (5), R (3.2)
CLDND1	9	10	1	9	AP- (1)	

CLK1	14	65	6	17	AP- (1), AP+ (7.1)	D (6.2), D (6.2)
CLK2	16	35	0	15	ES+ (5.2)	B (7), D (7), D (6), D (6)
CLN3	28	20	1	6	AT+ (14)	t (8), t (10.1), t (15), t (8), B (16.1), t (16.1)
CMAS	56	60	2	0	ES+ (6)	
CNOT2	17	7	1	10	AP- (1.3)	
CNOT8	17	19	0	16	ES+ (6)	
COA5	17	21	2	3	AT+ (3)	
COMMD7	26	16	3	2	ES+ (2.1)	
COX19	6	17	2	0	AT- (13)	
COX7A2	156	155	1	3	AP+ (2.1)	
CPSF4	30	53	8	45	ES- (4), AP+ (2)	z (3), z (3)
CRB3	9	14	2	11	AT+ (4.2)	
CROT	25	28	4	12	AT+ (5.2)	D (15), S (14), B (15), M (20), A (12), B (15), R (20), R (14), D (15), s (15), D (17), B (15), B (17)
CRYZ	17	16	2	13	ES+ (8)	B (8), B (4), R (4), R (8)
CSDE1	1009	1040	9	94	ES+ (4)	R (4), D (4)
CUTC	13	6	1	0	ES+ (7)	
CYFIP1	19	11	0	7	ES+ (29.2, 30.1)	
DCAF4	21	15	1	5	ES- (4.1), ES+ (5, 6)	
DDB1	130	115	2	70	AP+ (25.1)	R (21), R (3.2), R (21), R (12), R (3.2), R (12)
DDRGK1	14	24	0	0	AT- (7.3)	
DDX11	27	14	0	8	PS+	M (13), R (13), D (3.1)
DDX20	8	7	2	0	ES- (5.2)	
DECR1	35	42	10	0	ES+ (4.1, 4.2), ES+ (4.1), PS+	
DIABLO	12	18	2	2	ES- (2.2, 2.3), ES- (2.2)	
DNM1L	24	13	1	10	ES- (17, 18)	D (16), R (1), R (9), M (1), D (13), D (17), R (1), R (13), r (16), r (18), D (16), R (15), r (16), R (7), D (18), R (15), r (17)
DNTTIP2	14	27	1	0	ES+ (3)	
DPAGT1	29	18	1	13	ES- (9.2), AP- (2.2)	R (2.3), t (4.2), D (6), t (2.3), T (3), R (7), R (3), t (3), M (3), R (9.3), T (9.3), D (2.3), t (9.1), T (7), T (6), T (2.3), R (3), T (2.3), T (7), t (7), t (7), D (7)
DPM1	100	101	5	73	ES- (7.1, 7.2)	
DPP7	31	50	1	16	ES+ (1.2), PS-	
DSN1	19	11	2	15	ES+ (3.1, 3.2, 3.3)	
DSP	86	132	5	41	ES+ (23.2)	o (22), s (23.1), R (23.1)
DUSP11	11	46	4	8	AT+ (6)	D (4), D (4)
DUT	37	16	3	17	AP- (3.1)	
DYNC1I2	34	29	2	11	ES- (20.1), AP+ (1)	
EED	10	9	1	9	AP+ (10.1)	
EIF1AD	20	30	1	0	ES+ (2.1, 2.2), ES+ (2.1)	
EIF2B4	21	30	2	3	AP+ (1.3)	
EIF4A2	97	193	12	54	ES+ (4)	R (3), M (3), D (3)
EIF5	104	235	9	0	AP+ (3.1)	
EIF5A	168	159	7	33	AT+ (5.2)	
ELK1	19	16	1	5	ES+ (4.2, 5.1, 5.2, 5.3, 6.1)	D (5.1), D (5.2), D (5.1), r (5.1), r (5.1), D (5.1), R (5.2), r (5.1)
ENY2	21	19	6	7	ES- (5)	
EPN3	19	39	2	2	AP- (1.1)	B (2.2), D (2.1), D (2.2), B (2.1), D (2.1), D (2.2), B (2.2), B (2.1), B (2.1), D (2.1)
ERBB2IP	19	8	1	11	ES- (24.1, 24.2, 24.3)	
ERBB3	47	30	6	21	AT+ (5.2)	D (4), T (1.2), D (16), D (17.2), D (21), s (7), g (13), s (16), D (17.2), D (9), s (17.2), D (10), D (19.1), s (16), D (8), s (7), g (16), s (17.1), g (5.1), D (6), s (9), s (10), s (9), s (4), s (6), s (8), D (10), s (15), D (20.2), D (16), D (9), s (17.1), g (11), D (15), g (14), s (8), D (15), D (9), D (8), A (23), g (13), D (1.2), D (8), g (17.2), D (7), p (1.1), t (19.1), D (20.2), D (8), D (17.1), s (15), D (7), B (21), s (8), g (9), g (14), s (8), s (15), D (17.1), D (15), D (1.1), D (15), s (17.2), T (19.1), s (10), s (9), s (15), g (15)
ESRP1	59	27	0	31	ES+ (15)	
ETFA	236	189	2	55	ES+ (3)	
EXOC1	17	21	1	13	ES- (11)	
EXOC3	16	19	1	3	ES- (10.2)	

EXOC5	22	17	0	9	ES+ (4)	o (2)
EXOSC1	33	41	2	17	ES+ (3)	z (3), D (3)
EZR	97	75	4	0	AP+ (2.1)	
FAM122B	38	24	2	10	AP- (1.1)	
FAM134A	18	17	1	20	AP- (1)	t (5), t (2.2), t (1)
FAM136A	65	67	7	22	ES- (1.3), PS+	
FAM185A	6	8	1	0	ES- (1.4)	
FAM3A	11	10	1	5	ES- (7.1, 7.2), PS+	s (5.3), D (5.3)
FAM45A	22	22	0	0	PS+	
FAM73B	10	13	0	0	PS-	
FASTK	22	31	5	4	ES+ (5.9), ES- (5.6)	
FBXO25	8	8	1	0	ES- (5)	
FBXW7	8	15	1	10	AP- (1)	
FCHSD1	6	10	2	6	AP- (1)	
FKBP3	69	61	3	0	ES- (3), PS+	
FXYD3	46	123	3	8	ES- (4), AT+ (8.4)	T (8.3), D (8.3), t (8.1)
FZD6	18	22	2	2	AP+ (2.1)	D (6.1), s (5), D (3.4), p (3.3), T (3.3), T (6.1), g (3.4), T (6.1), s (3.4), D (5), D (5), t (6.1), s (3.3), D (6.1), D (3.3), t (6.1), t (6.1), s (5), D (3.3), D (3.3), D (3.3), s (5), t (6.1), T (6.1), D (5), t (6.1), t (6.1), D (6.1), D (3.3)
GCA	13	6	2	8	AP- (2)	
GCFC1	9	15	1	6	AT+ (16)	
GCH1	18	26	2	0	ES+ (6.2, 6.3, 6.4, 6.5, 6.6)	
GDI2	245	149	2	19	ES+ (7)	
GEMIN2	20	10	0	6	PS+	
GEMIN6	16	9	4	12	ES- (5.1, 5.2)	
GLB1	26	19	1	10	AP- (2)	g (4.2), D (1), p (1)
GLTP	28	58	1	2	ES- (3.2)	
GMPPA	22	15	1	9	ES- (2.1)	
GNAS	990	1513	3	195	AP+ (1)	
GNB2L1	714	673	21	80	AP+ (7.1)	
GNS	55	57	3	12	AP- (1.1)	D (1.3), D (1.2), m (1.3), D (1.3), m (1.3), p (1.2)
GPATCH4	25	35	0	29	PS-	R (2.2), D (2.2)
GPI	125	121	1	25	AP- (1)	
GRB7	5	14	2	8	AP- (1)	
GRHL1	11	13	0	5	PS+	N (1), R (1)
GRINL1A	7	9	0	0	AP+ (13.1)	
GRK6	25	13	1	8	AT+ (13.2)	R (17.2), l (17.2), R (17.2), l (17.2), l (17.2), D (13.1), R (13.1), R (17.2)
GSK3B	18	6	1	10	ES- (9)	
GSN	37	31	4	0	AP+ (7)	
GSS	30	44	3	6	AP- (1)	m (5.3), m (5.3), R (5.3), B (5.3)
GSTT1	9	20	2	12	ES+ (4)	
GUSB	30	27	2	6	ES+ (3.1, 3.2, 4.1, 4.2), ES+ (4.1, 4.2), PS+	g (3.2)
H2AFY	110	69	1	13	ES+ (7)	D (6.3), r (5), D (3), r (5), R (5), R (3), R (5), D (6.3)
H3F3A	438	221	2	6	AT+ (4.2)	
HAGHL	10	13	1	7	ES+ (5.4), PS-	
HDLBP	57	41	1	2	AP- (2)	D (11), D (10), D (10), A (11)
HERC4	22	13	2	9	AP+ (1)	D (28), A (28), D (21), R (21)
HMBS	28	14	1	7	AT+ (11.2)	
HMGN1	294	302	11	90	AP- (8.1)	
HMOX2	26	30	3	0	AP- (2)	
HN1	87	89	2	5	ES- (2)	
HNRNPA1	217	129	24	103	AT+ (11.5), PS+	
HNRNPAB	267	130	1	20	ES+ (5.3)	
HNRNPC	192	94	36	13	ES+ (2.2, 2.4), ES+ (2.4)	
HNRNPD	83	27	4	10	AT+ (8.2)	D (3), D (3)
HNRNPH1	154	213	40	137	ES- (1.4), ES- (2), ES+ (5), PS+	z (4), D (1.5), D (1.5), D (4), R (10.1), z (10.1), R (6), D (8), z (6), D (8)
HNRNPL	153	127	2	44	AP- (1)	D (3.2), D (3.2)
HNRNPU	142	97	4	50	ES- (1.4)	
HNRPDL	52	31	13	29	ES+ (6), ES- (8), PS+	R (6), R (6), R (6), R (6)
HNRPLL	15	11	1	11	AT+ (6.3)	D (3), z (3), D (9), D (9)
HRAS	23	55	2	9	ES- (7.2)	D (7.1), l (7.1), R (7.1), B (7.1), R (7.1), M

						(7.1), l (7.1), l (7.1), R (7.1), l (7.1)
HSP90AA1	1822	2264	6	51	AT+ (11.2)	
HTATSF1	51	63	1	3	AP- (2.1)	D (10.1), R (10.1)
ICMT	26	19	1	7	AT- (3)	t (2), D (5), D (4), t (6), R (4), R (6), t (5), t (4), t (5), t (5), D (6)
IKBIP	5	24	0	9	ES+ (2)	
IL6ST	7	8	0	0	ES+ (12)	
IMPDH1	42	38	1	33	ES+ (4.2)	
INCENP	21	10	1	19	ES+ (11)	
INF2	27	29	3	9	AP- (1)	B (20), R (8), o (18), R (2), D (8), D (2), D (20)
INO80E	10	8	0	0	ES- (6.1)	
IP6K2	14	27	7	17	ES+ (11.4, 11.5), AT+ (15)	R (14)
IRF3	33	50	4	2	AP+ (2.1)	D (6), R (5), R (5), s (6)
ISG20L2	24	26	0	0	ES+ (1.2)	
IVNS1ABP	75	33	2	34	ES- (9)	D (3), D (3), R (7), D (7), D (7), r (7)
KAT5	15	14	1	10	ES- (4)	
KBTBD4	16	19	1	13	AP+ (2.1)	D (2.5), D (2.5)
KDM2B	9	10	0	9	AP+ (16)	
KDM5B	28	36	1	13	ES- (6)	
KIAA0494	26	27	5	17	ES+ (7)	D (10), D (11), c (11), D (10), M (11), D (11)
KIAA0907	13	34	0	4	PS+	
KIAA1310	13	21	2	7	ES+ (21.1), ES+ (3.1), AT- (9), PS-	
KIAA1715	6	5	1	0	ES- (12)	
KIDINS220	14	14	1	7	ES+ (27)	D (18), T (18)
KIF23	29	9	1	14	ES+ (9)	
KIFC2	12	17	4	9	ES+ (16.3), PS-	D (11)
KLC1	35	33	2	18	ES- (15), ES+ (14.1), ES+ (13.3)	
KLHL12	21	35	1	21	AP+ (7.1)	R (10), R (10), D (3), D (5), D (3)
LDB1	21	78	2	30	AP- (1)	
LETMD1	27	27	3	12	ES+ (2, 3.2), ES+ (3.2), ES+ (3.2, 4.1, 4.2), PS+	s (1.1), R (2), T (5), t (4.1), R (1.1), D (5), D (2), T (1.1), D (1.1)
LGALS3	299	224	5	8	AT+ (6)	M (7)
LGALS8	7	8	2	0	ES+ (11)	
LMO7	10	8	1	9	ES+ (12)	
LRR1	29	26	2	6	ES- (4, 5), PS+	
LRWD1	19	23	0	0	PS+	
LSM1	34	44	6	37	ES+ (4)	
LSM14A	42	24	7	12	ES+ (10)	
LYPLA1	48	29	1	6	AT+ (7.3)	D (10), D (10), A (10), A (10)
LYRM1	19	11	1	0	AP- (1)	
MADD	15	13	2	6	ES- (25.2)	
MAGED2	58	56	5	2	ES- (14.2)	
MAGOH	38	53	6	23	ES+ (3)	
MANBAL	14	28	3	0	AP+ (2.2)	
RBM42	39	62	2	30	ES- (5, 6.1)	
MAP3K3	16	8	1	11	ES- (3)	
MAP7	43	14	1	14	ES+ (9)	o (7)
MAPK9	21	13	0	4	PS+	
MAPKAPK2	47	40	1	16	ES- (10.1)	R (10.2), R (10.2), D (10.2), M (10.2), R (9)
MARCH7	26	26	0	8	ES+ (12)	
MARK2	22	13	0	12	ES+ (19)	
MAST4	22	6	1	0	AP+ (10)	
MBD1	12	26	1	19	ES+ (18.1)	R (7), z (8), z (13), z (7), R (13), R (8), D (15), r (15)
MBIP	13	18	1	13	ES+ (7)	M (4), R (4), R (7), D (7), D (9.2), R (9.2)
MBTPS1	47	24	1	0	AP+ (12.1)	
MCFD2	39	57	1	0	AP- (1)	
MCM7	214	102	4	53	AP+ (2)	
MDH1	121	92	1	25	ES- (7)	B (6), R (6), R (4), B (7), B (6), B (7), R (6), B (6), B (6)
MDM2	30	117	6	68	AP- (1)	M (9), z (13.5), R (10), R (13.6), R (2.1), z (12), R (10), R (12), R (8.2), M (13.5), R (2.1), m (9), R (9), R (12), R (12), M (9), R (8.2), D (2.1), D (10), R (10), R (9), M (13.6), S (2.1)
MDM4	8	13	1	3	ES+ (11)	
METTL11A	36	32	3	21	ES- (5.2)	

METTL22	6	15	1	0	ES+ (4)	
MFSD10	24	39	2	9	ES+ (5.1), PS+	t (12.3), t (9), t (10), t (9)
MGAT4B	51	81	5	30	AP+ (2.1)	D (1), D (1), t (1), T (1)
MGEA5	30	45	4	32	AT+ (12.2)	m (11), R (11), s (16), R (14.1), m (14.1), D (16)
MIA3	10	12	1	7	ES+ (15, 16)	g (4.2), g (4.1), o (12.3), T (8.1), D (8.1), D (1), i (6), A (2), D (2), R (6), o (4.1), D (1), p (1), T (1)
MICALL2	12	14	0	5	PS+	D (1.1), z (1.1)
MMAB	15	9	1	11	ES- (5)	
MMADHC	71	86	1	10	ES- (7)	
MMS19	29	24	1	15	ES+ (17.1)	
MOCS2	16	13	1	0	ES+ (5)	
MORF4L2	97	110	16	1	ES+ (4, 5.1, 5.2), AP- (1), AP+ (3.1), AP+ (6.1)	
MRPL10	37	64	2	14	ES- (2.2)	
MRPL33	100	150	8	2	AT- (8)	
MRPL35	23	20	4	12	ES+ (4.2, 4.3), AT+ (4.4)	
MRPL52	14	22	6	9	ES- (1.2), ES+ (4.1), ES+ (3), AP- (1.1)	
MRPL55	13	10	0	1	ES+ (2.2), ES+ (2.2)	
MRPS18C	22	23	9	16	ES+ (3)	
MRPS27	37	21	0	15	ES+ (1.2)	o (12)
MRS2	23	25	1	13	ES+ (3, 4)	B (1), B (11.1), T (1), T (11.1)
MST4	31	28	5	26	ES+ (8)	D (3), D (3), D (3), B (3)
MTA1	62	45	5	24	ES- (4)	D (1), D (1)
MTFR1	32	14	4	1	AP- (2)	
MTHFSD	6	8	1	0	ES- (6)	
MTMR11	8	5	1	0	AP+ (1)	
MTMR14	25	16	0	8	AP+ (4), PS+	g (19)
MTRR	9	11	1	0	AP- (1.2)	
MTSS1L	33	44	2	10	ES+ (7)	o (6)
MYO19	17	8	4	10	AP+ (1.1)	
N4BP2L2	11	8	2	8	AT+ (10)	
NAA25	18	20	1	0	ES- (17), PS+	
NAA30	9	17	0	12	AP- (1)	
NAA60	23	29	1	0	ES- (4)	
NAE1	40	26	4	46	ES- (2)	
NAP1L1	144	109	1	17	ES- (13.3), AT+ (13.6)	B (14.1), l (14.1)
NASP	54	9	2	16	ES- (4)	
NCBP2	63	114	1	6	ES- (1.2)	D (3.2), D (3.2), B (3.2), R (3.2)
NCDN	15	17	2	5	ES- (1.2), AP- (1.1)	
NCOR1	17	7	1	10	ES- (20.1)	
NCOR2	24	9	1	13	ES- (23.1)	
NFE2L1	55	84	7	44	ES- (5), AP- (1)	D (2)
NKTR	12	11	0	8	PS+	r (15), D (15), R (15), R (15), D (2), R (2)
NME1	122	170	10	4	AT+ (6.3)	
NME2	72	89	5	1	ES- (8.3)	
NME4	97	89	7	16	AT+ (14)	D (5), B (5)
NOC4L	15	28	1	10	AP+ (10.1)	t (11), t (9), t (12)
NR2C2AP	27	29	2	14	ES- (3)	
NT5C3	24	17	1	11	AT+ (10.2)	m (11), B (11)
NUB1	14	18	0	6	PS-	D (15), D (14.1), R (14.1), R (13), D (14.1), M (14.1), D (16), M (13), D (13), D (15), R (16)
NUDT16L1	17	24	1	11	ES- (2.2)	D (2.1), R (2.1)
NUP160	25	14	3	14	ES- (3.2)	
NUP54	25	16	0	16	ES+ (12), ES+ (6)	R (1), m (1)
NUPL1	15	24	8	19	ES+ (5), AT- (19)	R (1.1), D (1.1)
NXF1	16	131	3	0	ES- (11.2), PS+	
OCIAD1	38	32	1	12	ES+ (3, 4)	R (3), D (3)
OCIAD2	24	35	3	8	ES- (6)	D (2), R (2)
ODF2	39	25	0	12	ES+ (10), ES+ (9.2, 10)	o (18)
ORC4	13	11	2	2	ES+ (2.2), AP+ (2.1)	
OS9	90	94	1	20	ES- (7.2, 7.3)	g (6)
OSBPL9	25	14	2	14	AP- (4)	D (4), z (4)
P4HA2	11	20	1	5	ES- (2.2)	D (12)
PA2G4	181	315	10	0	AT+ (13.2)	
PABPC1L	14	10	0	5	PS+	D (11), D (1), D (1), D (2), D (4), D (4), R (11),

						z (2)
PABPN1	18	29	12	24	ES- (5.2)	D (1.3), R (1.1), o (1.1), D (1.3), R (1.3), M (5.1), R (5.1), R (1.1), R (1.3)
PAPOLA	79	54	5	25	AT+ (7.2), AT- (9.4)	R (10), r (14), r (14), D (18), B (8), D (15), R (19), D (14), r (21), R (16), r (15), M (18), r (15), A (21), R (16), M (15), S (16), S (16), r (21), D (8), R (13), S (10), D (14), B (9.1), D (15), S (19), S (13), D (9.1), A (21)
PARP6	23	37	4	3	ES- (17), ES- (19)	
PDE8A	40	12	0	9	PS+	D (9), M (9)
PDLIM5	19	5	6	8	AT+ (4)	
PEX5	13	19	1	9	ES+ (9)	
PFDN1	34	30	2	18	ES+ (2)	
PFKM	19	13	1	8	ES+ (16.3), ES+ (16.2, 16.3)	B (16.3), B (16.3), B (16.3)
PHC1	5	5	0	0	AP- (1)	
PI4KB	39	59	1	13	ES+ (5)	D (4)
PICALM	64	40	2	25	ES+ (20), ES+ (19, 20)	R (21), S (21)
PIGF	10	9	4	9	ES+ (6)	
PIGT	60	82	2	32	ES+ (2.1, 2.2, 3)	D (4), s (4), R (1), g (3), T (1)
PILRB	29	31	2	4	ES+ (10.1, 10.2)	D (15), g (14.2), D (13), D (13), T (15), T (13), D (13), t (15)
PIP5K1A	34	54	0	24	ES- (1.2)	r (5), D (5), s (5), D (5)
PITRM1	80	106	7	2	AP- (1)	
PKMYT1	26	20	4	5	AP+ (1)	
PLA2G15	10	12	0	10	ES+ (5.1)	D (6), D (6), A (6), g (6), g (6), A (5.1), A (6), D (5.1), g (6)
PLD3	24	29	3	2	ES+ (4)	g (8.2)
PLXNB2	37	42	2	1	ES+ (3.2)	D (9), g (21), D (19), D (22), D (15), g (15), D (7.1), g (14), D (23), D (4), S (22), D (7.2), D (18), D (18), g (8), g (21), g (21), D (15), D (7.1), g (15), T (23), g (16), D (7.2), D (19), g (20), t (23), T (4)
PMEPA1	40	36	3	15	AP+ (2.1)	D (3), T (3), t (5)
PMPCA	45	59	4	25	AT+ (6.2)	
PMPCB	56	34	5	24	ES- (12.2), PS+	m (3.1), m (3.1), R (3.1), R (3.1), A (3.1)
PNISR	16	17	0	0	PS+	
PNPO	40	83	3	19	ES+ (4)	R (3), B (3)
PNPT1	25	12	0	11	PS+	
POFUT2	8	18	1	9	ES- (8.4)	
POLR2D	29	43	6	3	AP- (1)	
POLR3D	10	14	0	0	PS+	
POM121	14	26	1	18	AP- (1)	R (5), t (5), D (5), D (5), R (5), R (5), R (5)
PPIL2	14	10	1	0	ES+ (22.3)	
PPIL3	10	8	1	9	ES- (1.3)	
PPP1R7	12	16	1	15	ES+ (2)	z (11), D (11)
PPP2R5C	31	11	5	9	AT+ (3)	M (19)
PQBP1	35	25	2	9	AT+ (4)	R (6), R (6), R (6), R (6), D (3), o (5), D (3), R (6), R (6)
PRDX2	209	311	1	6	AT- (3.2)	
PREB	42	44	1	9	ES- (6)	T (1.3), D (1.3)
PREPL	13	17	2	1	AT+ (15.2)	
PRMT1	111	120	1	7	ES- (11.2)	
PROM2	25	42	0	7	PS+	D (12), T (12)
PROSER1	26	28	1	15	ES+ (2)	
PRPF3	38	66	2	31	ES- (4)	D (2), M (2)
PRPF38B	26	33	3	22	ES- (2)	o (8)
PRRC2C	44	26	0	34	ES- (34)	
PRUNE	20	14	3	25	ES+ (4), ES+ (4, 5.2, 6), AP- (1)	R (6), m (3), R (4), m (4), R (3), R (4), B (4), M (4), m (6), m (4), R (3), m (3)
PSMA1	73	99	1	14	ME+ (8.2, 9)	
PSMD13	49	77	2	23	ES- (3.2)	D (8)
PTGES3	413	233	6	53	ES+ (6)	D (2), D (2)
PTPMT1	39	35	0	7	ES- (1.2)	
PTPRF	65	27	2	4	ES+ (12)	m (13), R (12), D (12), T (3.2), D (13), D (3.2)
PTTG1	47	41	2	0	AP+ (2.1)	
RAB1A	107	99	4	21	ES+ (6, 7)	R (6), R (7)
RAB5C	82	103	4	0	ES- (2)	
RABL5	21	19	0	9	PS-	R (4), R (1)

RAD1	11	7	4	0	ES+ (7)	
RAD17	11	9	0	10	ES+ (5)	
RAD21	159	97	1	45	AP+ (12.1)	z (6), R (8), S (9), R (8), S (8), S (11.2), R (9), D (8), z (11.2), S (6)
RAD51C	68	54	15	17	ES+ (7), AT- (10)	M (10)
RAE1	66	105	1	4	AT+ (12.2)	
RALGAPB	13	18	3	8	ES+ (25.1)	D (23), D (23)
RANBP2	24	23	4	16	ES+ (20.1)	D (20.1), N (20.1), M (20.1), D (20.1), S (20.1), z (20.1), A (20.1), M (20.1), R (20.1), z (20.1), z (20.1), z (20.1), z (20.1), z (20.1), z (20.1), z (20.1), z (20.1), D (20.1)
RANGAP1	31	36	3	1	AP+ (6.1)	
RBBP6	17	34	1	18	ES+ (16)	z (8), R (17), R (8), z (6), S (18), R (6), R (18), R (17)
RBL2	23	13	1	22	AP+ (6)	
RBM3	73	98	3	17	ES- (5.2), AP+ (2.1)	D (2.2), D (2.2)
RBM39	62	89	5	37	ES- (4)	D (13), z (16), R (17), D (13), R (17), R (16)
RBMX	111	39	0	24	PS+	D (2), D (2)
RC3H1	6	9	2	0	ES+ (20.1)	
RCC1	64	46	1	36	ES- (7)	
RCCD1	15	7	1	10	AP+ (1)	
REPIN1	43	34	2	27	ES+ (4.2), AP- (5.1)	
REPS1	13	9	1	10	ES+ (12.2, 12.3, 13), ES+ (12.3, 13)	
RETSAT	12	121	0	7	PS+	M (2)
RFC5	31	27	2	7	ES+ (2.2)	
RFWD2	22	5	1	8	ES+ (9)	
RHBDD2	46	146	4	29	ES+ (2)	t (3), t (3), t (1)
RIF1	15	9	2	11	ES+ (31)	
RINT1	30	40	5	0	ES- (3)	
RPAIN	9	10	2	9	ES+ (6.1)	
RPE	29	18	0	11	ES- (8)	m (1.1), R (10.2), A (1.1), m (1.1), B (1.1), R (1.1), R (1.1)
RPL14	378	413	4	5	ES- (1.2, 1.3)	
RPL21	662	961	1	0	ES- (1.4)	
RPL22L1	45	28	9	7	ES- (3.1), PS+	
RPL34	306	447	12	0	AT- (8)	
RPL7	780	931	5	4	AT+ (7.2)	
RPLP0	1904	2924	57	557	AP+ (6.1)	
RPS10	983	1480	1	14	ES+ (6)	
RPS19BP1	37	42	2	0	ES- (3)	
RPS21	889	692	397	15	AT+ (3.11)	
RPS23	169	263	1	0	ES- (3.3)	
RPS27L	24	82	8	13	AT+ (5.2)	
RPS29	1510	462	1080	22	AT+ (3), AT+ (4)	
RPS6KA4	17	20	0	4	PS-	
RUNX1	10	5	1	6	ES+ (8)	
RWDD4	14	13	3	5	ES+ (3.1)	D (2), R (2)
S100A14	135	145	2	0	AP+ (3)	
SAE1	98	73	0	5	ES+ (4)	
SAP30BP	38	48	2	7	ES+ (3)	
SAT2	6	28	1	10	ES- (1.2)	
SBF1	11	19	1	14	ES+ (29)	
SCAF4	9	13	2	10	ES+ (3)	
SCAND1	13	23	5	0	ES- (3.2)	
SCARB1	50	24	1	7	AT+ (13.4)	T (1), D (13.2), D (1), g (5), T (13.2), g (5), t (13.2), t (1)
SDCBP	16	46	1	8	ES- (3.2)	D (6.2), z (6.2)
SEC14L1	12	12	4	0	AP+ (1), PS-	
SEC16A	34	29	6	15	AT+ (27.3)	R (1.3), R (23.3), R (1.3), R (23.3)
SEMA3B	13	17	0	0	PS-	
SENP6	18	19	1	6	ES+ (8)	A (22), A (21), p (22), A (25), R (18.1), p (25), R (18.1), p (21)
SEPT2	51	34	2	8	ES- (8)	
SERF2	45	40	29	1	ES+ (3.3, 3.4), AT+ (7.2)	
SETD8	30	28	0	0	PS-	
SF3B1	78	73	3	15	ES- (4.2, 4.3)	D (12), D (7), R (7), R (12)
SFSWAP	16	13	0	6	ES+ (2.2), PS+	

SFXN4	23	12	2	5	AP- (1)	
SH3GL1	33	23	0	5	ES+ (4)	D (2), M (2), R (3), s (3)
SH3YL1	11	12	6	4	ES+ (19)	D (20), A (20)
SHARPIN	15	20	0	0	PS-	
SHC1	22	77	12	38	AP- (1), AP+ (2.1)	D (12), D (12)
SHFM1	171	92	4	4	AT+ (4.2), AT+ (7)	
SHMT1	23	8	0	12	ES+ (12)	
SIDT2	13	21	2	12	ES+ (11)	D (1.1), t (25.1), g (20), g (4), g (18), D (22), g (21), T (19), T (1.1), D (22), g (1.2), t (19), D (9), D (25.3), g (3), T (22), t (22), g (1.2), D (20), g (3), T (25.3), D (19), T (9), t (16), T (22), t (9), t (20), D (17), g (18), T (20), T (17), t (21)
SKA3	20	10	0	3	PS+	
SLC1A5	115	87	1	8	AP- (2)	g (5), t (6)
SLC20A1	41	37	2	16	AP+ (6.1)	t (5), t (3), D (3), D (4), t (2), T (4), T (2), T (2), D (2), T (2), D (2), t (2), D (2), t (2), T (3)
SLC25A15	22	21	0	14	PS-	t (3.3), t (3.3)
SLC25A25	10	31	9	27	AP- (1), AP+ (3.1)	D (5), c (5), D (3.1), D (5), D (6), T (3.1), D (6), D (3.1), D (5), D (3.1)
SLC25A29	23	15	2	3	ES+ (3.2, 3.3, 3.4, 3.5)	t (1), t (4), t (1), t (4)
SLC25A3	253	347	3	31	ES- (2.2), AT+ (4.2)	t (6), t (8), T (5), T (7), B (5), D (8), R (6), T (6), D (4.1), t (6), D (6), T (8), B (7), T (4.1), t (5), t (5), M (8), t (6)
SLC26A6	7	9	1	0	AP- (2.1)	
SLC2A8	11	38	1	11	ES- (5)	M (1), D (6), D (3), T (4), T (2), t (3), t (2), D (1), t (6), R (4), t (3), D (4), t (2), T (3), t (5), M (5), D (5), t (3), t (3), T (3), t (2), T (5), D (3), D (1), D (2), T (1), t (4), t (2), D (5), T (5)
SLC30A6	17	11	1	13	ES- (5)	t (10), D (8), T (1), t (8), T (3), t (4), T (6), t (3), T (10), T (13), T (8), D (3), D (6), D (10), D (1), D (13)
SLC35A2	13	28	1	10	ES- (5.1)	t (5.1), t (5.1), t (4.1), t (5.1), t (5.1), t (5.1)
SLC35C2	23	18	1	13	ES+ (8.1)	t (3.5), t (4), g (5), t (6), t (5)
SLC35E2B	10	8	1	0	ES- (2)	
SLC35F5	19	14	2	7	AT+ (7.2)	D (8), t (9), t (7.1), t (13), t (12), t (8), t (14), t (10), t (11)
SLC38A1	94	28	1	1	ES- (1.2, 3), ES- (3)	t (18)
SLC38A2	118	64	4	19	ES+ (4), PS+	t (4), t (6), t (4)
SLC3A2	108	288	19	98	ES+ (2, 3, 4.1, 4.2), AP- (1)	D (5.2), T (5.2), g (9), D (5.3), g (10), g (10), t (5.3)
SLC6A6	17	7	2	9	AT+ (6.2)	
SLCO4A1	8	5	1	0	AP- (1)	
SLMO2	146	206	16	57	ES+ (3.2), ES+ (3.1, 3.2)	R (1), D (1)
SMARCA4	51	19	1	20	ES- (36.1)	
SMARCB1	48	37	4	21	ES+ (2.2)	S (3), R (3)
SMARCD1	26	34	2	16	ES+ (12)	R (5), R (4), R (4), R (5), o (11)
SMARCD2	164	148	2	183	AP+ (1.2)	
SMC6	16	8	1	10	ES- (9)	
SMEK2	28	17	3	18	ES+ (12)	
SNRNP40	29	17	2	13	ES- (10)	
SNRNP70	40	52	1	12	ES+ (8.1, 8.2)	D (5), D (5)
SNRPB	76	66	2	9	AT+ (7.4)	S (6), R (6)
SNRPG	87	60	5	6	AP+ (2.1), AP+ (2.2)	
SPDEF	86	53	1	16	ES+ (4)	
SQSTM1	38	92	8	3	AP- (1), AP+ (5.1)	R (3), S (3), R (3), R (3), D (3), R (3), D (3), R (3)
SRPK2	50	8	2	13	AP+ (1)	
SRSF11	33	36	1	4	AP- (1)	
SRSF2	129	131	14	0	ES+ (2.7), ES- (2.4), PS+	
SRSF3	124	77	8	33	ES- (4.1), AT+ (4.2)	R (5), D (5)
SRSF7	63	54	10	30	ES- (4.3, 4.4), PS+	D (3), R (4.6), D (3), r (4.6)
SS18L1	15	24	0	7	PS-	R (1), R (1)
SSFA2	16	11	2	2	AP- (1)	
SSH3	38	31	2	0	ES+ (4.2)	
SSU72	18	8	2	12	AT+ (2.2)	
STAG2	43	19	2	7	AP+ (2), AP+ (4.1)	
STAG3L2	5	5	1	0	ES+ (6)	
STIP1	229	162	1	14	AT+ (6.2)	

STRAP	108	132	3	34	ES+ (3)	
STX10	21	19	4	11	ES- (7.2)	t (7.3), D (7.3)
STX16	59	106	6	51	ES- (7.1, 7.2)	D (1.5), T (1.5)
STX18	10	5	1	7	AP+ (2)	
SUGP1	11	11	3	1	AT+ (16)	R (14), D (14), M (10)
SULF2	92	117	17	81	ES+ (20)	
SUPT5H	41	81	2	18	AP- (1)	
SUPT7L	10	17	1	3	ES+ (2.3), ES+ (2.3, 2.4)	
SYF2	16	34	1	13	ES+ (3)	o (3)
SYPL1	147	152	3	35	ES- (6)	D (7), D (2.2), T (7), D (2.2), t (5)
SYTL1	9	34	1	3	ES- (8.8)	
SYTL2	59	34	8	35	ES+ (11.2)	D (2), D (2)
TAF1D	15	22	0	0	PS+	
TAF2	18	11	0	0	PS-	
TAF6	42	41	4	24	ES- (3)	
TARBP2	22	31	1	5	ES- (7.1)	D (3.2), R (3.2), S (3.2), R (3.2)
TBC1D14	14	21	1	16	AP+ (6)	D (10), R (10)
TBC1D7	21	19	1	11	ES+ (5)	R (3), D (3)
TCEB1	24	33	1	2	AP- (1.2)	
TCOF1	29	15	2	19	ES+ (7)	
TCTN3	19	15	2	15	ES+ (8)	T (14), t (14), D (1), D (14), T (1)
TFAM	21	28	2	8	ES+ (5)	R (5), N (5)
TFDP1	44	26	1	5	AP+ (1)	
THAP5	21	15	2	8	AP- (1)	D (4), D (1), o (4), z (1)
THOC3	85	96	3	6	AT+ (6.2)	
TIA1	17	11	2	11	ES- (8), AT+ (9.2)	D (11), z (7), D (11), D (7)
TIAL1	39	55	5	28	AT+ (5.2)	D (5.1), D (9), z (5.1), D (9)
TIMM50	22	26	0	0	PS+	
TIPIN	15	5	1	0	ES+ (4)	
TJP1	18	10	4	13	ES+ (23), ES- (30.2)	D (29), D (29)
TLE2	11	10	1	8	ES- (9.2), PS+	M (11), R (11), D (11)
TM2D3	12	22	1	6	AP+ (2.1)	t (4), D (1.1), p (1.1), t (5)
TMBIM4	15	9	1	9	AP+ (2.1)	
TMED2	172	164	9	55	AP+ (2.1)	T (1.1), D (1.1), D (1.2), R (1.2), R (1.1), R (1.1), p (1.1)
TMEM126B	18	21	1	12	ES+ (2.1), ES+ (2.1, 2.2, 4)	t (6.1), t (5.1), t (5.2), t (6.3)
TMEM134	16	40	2	11	ES- (2.2)	T (8), t (7), D (1), D (8), T (1), t (4.1)
TMEM59	71	80	2	10	AP- (1.1)	T (1.2), g (2.2), D (1.2), D (1.2), p (1.2)
TMEM63A	11	10	1	4	AP+ (9.1)	t (21), g (16), t (22), g (3), t (3), t (20), t (16), t (7), t (9.2), t (23), t (17), t (18), t (21)
TMEM70	13	14	5	9	ES- (3), PS+	t (2.1), t (4)
TMEM93	33	43	3	0	ES- (1.2)	
TMPO	65	26	19	37	ES+ (6, 7, 8), AT+ (4), AT- (10)	M (4), o (4)
TMUB2	15	17	2	12	ES+ (3, 4.1), ES+ (3)	
TNFRSF1A	37	27	1	7	AT+ (6)	D (7), D (8), D (2), M (11), T (8), s (7), R (10), s (5.1), R (10), D (5.1), t (8), T (2), D (11)
TOR2A	6	16	1	5	ES- (2)	
TPM1	68	47	2	20	ES- (12.2)	D (3.2), r (3.2)
TPX2	97	33	1	7	ES- (11)	
TRAPPC6B	22	27	0	9	PS+	
TRIM11	8	19	1	11	AT- (5.2)	
TRIP12	35	24	0	19	ES+ (19.1)	D (18), D (18)
TSC22D1	18	33	3	10	AP- (1.1)	
TSEN15	25	10	2	9	ES+ (5.1, 5.2)	
TSPAN31	15	16	3	8	AT+ (6.2)	T (2.2), T (2.1), g (3), t (1), g (5.2), g (5.2), t (2.2), D (2.1), D (2.2), t (2.1), g (5.2), T (3), D (3)
TTC31	10	11	0	3	PS-	
TTI1	21	11	1	0	ES+ (2)	
TTYH3	8	22	1	11	AP- (1)	
TUFT1	15	48	2	17	ES+ (3)	o (5)
TYMS	61	100	2	17	ES+ (4)	
UBB	471	612	0	5	ES- (2.2)	
UBE2A	41	69	10	44	ES+ (4.2)	
UBE2B	22	27	1	0	ES+ (3, 4)	
UBE2C	49	33	0	6	ES- (3.2)	
UBE2G2	28	44	0	14	PS-	

UBTF	33	28	1	5	AP+ (5.1)	R (10), N (10)
UBXN1	38	62	2	26	ES- (8.2)	R (3), R (3), B (7), D (7)
UBXN2A	5	5	1	0	AP- (3)	
UEVLD	11	8	1	9	ES- (3)	
UGDH	171	55	2	26	ES+ (4)	R (4), R (4), R (4), B (4), R (6), R (6), B (4)
UMPS	34	36	3	19	ES+ (3)	D (1), R (1), R (4.2), R (4.2)
UQCC	5	5	0	0	ES- (2)	
USMG5	87	111	1	0	ME- (2.2, 3)	
USP15	18	21	0	8	AP- (4)	D (1), A (10), A (22), D (1)
USP16	14	12	2	10	ES+ (8.1)	
USP8	41	32	6	22	ES+ (4), ES+ (13)	
VEGFA	14	19	2	13	ES+ (7.1, 7.2, 8.1, 8.2)	D (1), p (1)
VMP1	246	218	16	21	ES+ (4)	T (2), t (4), t (3), D (3), T (4), T (3), D (4), D (2)
VTI1B	40	39	1	0	ES+ (2, 3)	
WARS	57	49	2	28	ES+ (5)	D (5), R (5)
WBSCR22	63	49	6	9	AT+ (10.2)	
WDR37	11	10	0	5	AP- (3)	
WDR6	36	43	5	13	ES- (5.6), PS+	
WIPI1	9	10	2	10	ES+ (2)	
WIPI2	16	19	2	10	ES+ (12.2)	
XRCC3	18	23	5	0	AP- (1.1), AP+ (3.1)	
YIF1A	38	93	10	13	ES+ (2), AP- (1)	D (8.4), t (7), D (7), t (8.2), T (7), t (8.4), t (4.2), T (6), D (8.4), D (6), T (8.4), T (8.4), t (7)
YIPF1	11	20	0	4	PS-	T (8), t (7), T (4.2), t (9), R (8), D (4.2)
YME1L1	78	78	5	51	ES+ (5)	B (1)
YWHAZ	663	524	1	50	AP+ (6)	S (7.4), R (7.4)
YY1AP1	16	19	4	16	ES+ (12.1)	
ZBTB11	14	30	0	15	ES- (2.2)	z (4), z (8), z (9), D (2.3), z (8), z (10), z (7), z (8), z (10), z (4), z (8), z (7), z (10), z (6), z (5), z (10), z (5), z (7), z (9), D (2.3), z (7)
ZC3H15	49	58	2	13	AT+ (9.2)	
ZC3HAV1	14	13	1	7	AT+ (9.4)	t (10), D (10)
ZDHHC20	51	29	1	18	ES+ (14)	t (1), t (3)
ZDHHC4	23	37	1	0	AP- (1.2)	
ZDHHC7	47	75	8	29	ES- (4)	
ZMIZ1	22	14	3	17	AP- (5)	
ZMYM5	7	15	2	9	ES+ (5)	
ZNF195	8	17	0	9	ES+ (3)	
ZNF207	13	12	4	7	ES- (9)	
ZNF217	76	224	6	38	ES+ (6)	
ZNF385A	36	161	3	24	ES+ (7)	R (7), z (7)
ZNF398	6	6	1	0	AP+ (1)	
ZNF410	39	23	4	30	ES+ (3.2, 3.3), ES+ (3.3)	z (11), z (10), z (9.2)
ZNF706	15	28	2	0	AP+ (3.1)	
ZNF707	8	20	2	0	ES+ (7.2)	
ZNF789	7	11	1	8	ES+ (3)	

RPKM (Reads per kilobase per million reads) of control and cisplatin-treated MCF7 cells, their alt splice RPKM (RPKM level of transcripts with an alternate splice form), alt protein RPKM (RPKM level of alternative transcripts with splicing changes in their protein coding regions), and the type and exon location of splice events were indicated. The SpliceSeq software identified 717 events concerning 619 primary transcripts. A: active site; C: Ca2+ binding; P: nucl. phos. binding; m: metal binding; B: binding site; z: zinc-finger; N: DNA binding; r: cross-link; s: disulfide bond; g: glycosylation; l: lipid binding; i: intramembrane; t: trans membrane; o:coiled-coil; T: topological domain; p: signal peptide; M: short sequence motif; D: domain; S: site; R: region of interest; n: non-standard AA; ES: exon skip; AT: alternative terminator; AP: alternative promoter; PS: premature stop.
